# Natural products mitigate pyrethroid-induced male reproductive toxicity: a systematic review

**DOI:** 10.3389/fphar.2026.1933092

**Published:** 2026-10-26

**Authors:** Atefeh Babaei, Sajjad Moradi, Nima Azari-Dolatabad, Sajad Fakhri, Mohammad Hosein Farzaei, Javier Echeverría

**Affiliations:** 1 Pharmaceutical Sciences Research Center, Health Institute, Kermanshah University of Medical Sciences, Kermanshah, Iran; 2 Medicinal Plants Research Center, Maragheh University of Medical Sciences, Maragheh, Iran; 3 Department of Nutrition and Food Sciences, Maragheh University of Medical Sciences, Maragheh, Iran; 4 Department of Internal Medicine, Reproduction and Population Medicine, Faculty of Veterinary Medicine, Ghent University, Merelbeke, Belgium; 5 Department of Animal Science, University of Tennessee Institute of Agriculture and AgResearch, Knoxville, TN, United States; 6 Departamento de Ciencias del Ambiente, Facultad de Química y Biología, Universidad de Santiago de Chile, Santiago, Chile

**Keywords:** male, natural antioxidants, pyrethroids, reproductive toxicity, systematic review

## Abstract

**Background:**

Pyrethroids are among the most widely used pesticides worldwide and cause reproductive toxicity through oxidative stress, endocrine disruption, and testicular damage. Numerous studies have explored antioxidant-rich natural products and other bioactive compounds to mitigate the effects of pyrethroid exposure.

**Purpose:**

This systematic review comprehensively evaluates the evidence on the efficacy of natural products in mitigating pyrethroid-induced reproductive toxicity in experimental models.

**Methods:**

Following PRISMA guidelines, PubMed, Scopus, Web of Science, and Embase were searched from database inception to July 2025. The animal experimental studies evaluating natural products against pyrethroid-induced reproductive toxicity in adult male laboratory animals were included. Outcomes included sperm parameters, hormonal balance, oxidative stress markers, and testicular histopathology. Study quality and risk of bias were assessed using the ARRIVE guidelines and SYRCLE’s Risk of Bias tool for animal studies, respectively.

**Results and discussion:**

Thirty-three animal experimental studies were included in this review. Most indicate that pyrethroid exposure consistently decreased sperm count, motility, and testosterone levels, increased oxidative stress, and disrupted the hypothalamic-pituitary-gonadal (HPG) axis. The studies show that natural products such as sage (*Salvia officinalis*), rosemary (*Salvia rosmarinus*), onion (*Allium cepa*), ginseng (*Panax ginseng*), curcumin, quercetin, and resveratrol may ameliorate these effects.

**Conclusion:**

Proposed mechanisms of natural products include enhanced antioxidant defenses, hormonal regulation, DNA protection, and preservation of testicular, structure although direct evidence for these pathways remains limited and has largely been inferred from a subset of the included studies rather than established as generalizable mechanisms. Nanoparticle formulations may improve bioavailability and efficacy. Natural antioxidants show promise in reducing pyrethroid-induced reproductive toxicity in male experimental animals. Further standardized research and clinical trials are essential to establish translational potential.

**Systematic Review Registration:**

https://www.crd.york.ac.uk/PROSPERO/, identifier CRD42022348260.

## 1 Introduction

Infertility is a major global public health issue, affecting approximately one in six couples of reproductive age worldwide, with male factors contributing to nearly half of these cases. This underscores the urgent need for a comprehensive understanding of the etiologies and mitigating factors of male reproductive dysfunction (Agarwal et al., 2021). Chromosomal abnormalities, genetic mutations, and hormonal disorders are among the genetic factors associated with male infertility. Additionally, environmental factors, including exposure to toxins, heavy metals, and chemicals, can adversely affect sperm quality and quantity (Bhattacharya et al., 2024). In the modern era, humans are continuously exposed to a wide variety of chemical substances, primarily due to industrialization and lifestyle changes. This extensive exposure, particularly to pesticides, has emerged as a major public health concern (Sengupta and Banerjee, 2014).

Pyrethroids have gained widespread use in agricultural and domestic contexts because of their enhanced efficacy and reduced toxicity compared with predecessor organochlorine compounds (Yao and Wang, 2008). Among the top 10 pesticides used globally, pyrethroids account for over 30% of worldwide insecticide consumption and are widely dispersed in the environment (Fenner et al., 2013). Empirical studies have consistently detected pyrethroids and their metabolites in residential environments, food systems, and human biological samples (Barr et al., 2010). Cypermethrin, fenvalerate, permethrin, pyrethrin, pyrethrum, resmethrin, tetramethrin, and fenpropathrin are the most common pyrethroids (Ahamad and Kumar, 2023). The United States Environmental Protection Agency (EPA) has classified pyrethroids as Endocrine Disrupting Chemicals (EDCs) because they can mimic and potentially disrupt natural hormonal functions (Zhang et al., 2016; Ye and Liu, 2019). Pyrethroid exposure has been linked to reduced sperm count and motility, abnormal sperm morphology, altered reproductive hormone levels, oxidative stress, deoxyribonucleic acid (DNA) fragmentation, and testicular histopathology (Atef et al., 2020; Radi et al., 2021).

One of the most significantly affected systems is the hypothalamic-pituitary-gonadal (HPG) axis (Ye and Liu, 2019), which plays a critical role in regulating sexual hormone production and sexual maturation. Pyrethroids can disrupt mitochondrial function and increase free radical production. These highly reactive free radicals can damage sperm DNA, cellular membranes, and other cellular components (Atef et al., 2020; Radi et al., 2021). They can also decrease the body’s natural antioxidant levels and inhibit the activity of antioxidant enzymes such as superoxide dismutase (SOD), catalase (CAT), and glutathione peroxidase (GPx) (Ben Halima et al., 2014; Desai et al., 2015; Sharma et al., 2018), which play crucial roles in neutralizing free radicals.

Natural products, including medicinal plants and phytochemicals, are rich in antioxidants such as flavonoids, polyphenols, and carotenoids. These compounds may mitigate oxidative stress, restore hormone balance, and protect testicular structure in experimental models (Oda and El-Maddawy, 2012). Moreover, novel delivery systems, such as nanoparticles, may enhance bioavailability and therapeutic efficacy (Mohamed et al., 2023).

Ultimately, given the increasing use of agricultural pesticides and their side effects on human health and fertility, identifying and mitigating harmful reproductive effects using natural compounds could be a viable solution.

Although recent reviews have discussed insecticide-induced testicular injury and the potential protective effects of natural products (Darwish et al., 2025), a focused systematic synthesis of experimental evidence on natural products against pyrethroid-induced male reproductive toxicity remains limited. In particular, previous reviews have generally considered multiple insecticide classes and have combined mechanistic, human, and animal evidence. The present systematic review addresses this gap by systematically evaluating experimental studies in male animal models, with specific emphasis on sperm quality, reproductive hormones, oxidative stress, testicular histopathology, and molecular mechanisms associated with natural-product-mediated protection.

This systematic review synthesizes current evidence on the protective role of natural products against pyrethroid-induced male reproductive toxicity, identifies underlying mechanisms, and highlights research gaps requiring further exploration.

## 2 Methods

### 2.1 Literature search

This study was conducted in accordance with the Preferred Reporting Items for Systematic Reviews and Meta-Analyses (PRISMA) guidelines for systematic reviews (Moher et al., 2009; Page et al., 2021), and the study’s protocol was approved by the International Prospective Register of Systematic Reviews (PROSPERO; ID: CRD42022348260). A description of the population (adult male laboratory animals, including rats, mice, rabbits, and guinea pigs, of any eligible species or strain), intervention (natural products, including medicinal plants, plant extracts, essential/fixed oils, isolated phytochemicals, vitamins, and other naturally derived), comparator (Pyrethroid-exposed animals receiving no natural-product intervention), and outcome (sperm count, motility, viability, morphology, reproductive hormones, oxidative stress/antioxidant markers, testicular histopathology, apoptosis/DNA damage, and molecular reproductive outcomes) (PICO) framework is reported in Supplementary Table S1.

Initially, a comprehensive systematic search was conducted on Scopus, Web of Science, PubMed, and Embase. In the search strategy, we used medical subject headings (MeSHs), abstracts, and keywords. Search strategies, including the key terms and the queries for each database, are provided in Supplementary Table S2. Additional searches were conducted in Google Scholar and the reference lists of eligible articles. Searches covered publications from database inception to July 2025. Two co-authors (A.B. and S.M.) independently carried out the systematic assessment. Any discrepancies were resolved via discussions with other investigators (M. F. and J.E.).

### 2.2 Research question

The primary research question guiding this systematic review was: “What is the evidence from experimental animal studies regarding the protective effects of natural products against pyrethroid-induced male reproductive toxicity?”

### 2.3 Eligibility criteria

Two (A.B. and S.M.) investigators read the titles, abstracts, and full text of the publication as needed, and then selected eligible articles. Studies were selected for further assessment if they met the following inclusion and exclusion criteria:

Study design: Experimental animal studies (all animal studies). The Population (P) component of the PICO framework included adult male laboratory animals (rats, mice, rabbits, and guinea pigs), as defined by the respective studies. No restrictions were placed on the strain or species to ensure a comprehensive overview of the available evidence. Intervention: Pyrethroid exposure and/or natural product treatment. Outcomes: a) Sperm quality and quantity, b) Testicular tissue effects, c) Cellular and molecular fertility effects, d) Oxidative stress markers, and e) Effects of plant compounds on the above parameters.

We excluded case reports, letters, reviews, conference proceedings, editorials, non-English articles, and observational studies. Any conflicts during the study selection were resolved through discussion with another researcher (M.F. and J.E.).

### 2.4 Data extraction

Table 1 shows the main features of the included studies. All identified records were exported to EndNote 21 (Clarivate, Philadelphia, PA, USA) for reference management and duplicate removal. For each study, the first author, year of publication, study location, study design, total sample size, patient characteristics, Time of assay (days), target tissue, method of assessment, the effect of insecticide compared with the control group, the effect of insecticide and herbal medicine compared with the insecticide group, and main outcomes were recorded. Two researchers (A.B. and S.M.) independently extracted data from eligible articles to improve outcome reliability. Conflicts, if any, were resolved via consensus and discussion by a third author (M.F.).

**TABLE 1:** Risk of bias of the 33 included studies using SYRCLE’s Risk of Bias tool.

Number	Included studies	D1	D2	D3	D4	D5	D6	D7	D8	D9	D10
1	Abd-Ellaah et al., 2024	Y	U	U	U	U	U	U	Y	Y	U
2	Adeniyi et al., 2024	Y	U	U	U	U	U	U	Y	Y	Y
3	Ali Hasan and Al-Rikaby 2023	Y	U	U	U	U	U	U	Y	Y	Y
4	Chouabia et al., 2021	Y	U	U	U	U	U	U	Y	Y	Y
5	El-Demerdash et al., 2003	Y	U	U	U	U	U	U	Y	Y	Y
6	Herman et al., 2024	U	U	U	U	U	U	U	Y	Y	U
7	Khormi et al., 2025	Y	U	U	U	U	U	U	Y	Y	Y
8	Sharma et al., 2013	Y	U	U	U	U	U	U	Y	Y	Y
9	Sharma et al., 2014	U	U	U	U	U	U	U	Y	Y	U
10	Ubah et al., 2021.	U	U	U	U	U	U	U	Y	Y	Y
11	Vemo et al., 2017	Y	U	U	U	U	U	U	Y	Y	Y
12	Yousef et al., 2003	Y	U	U	U	U	U	U	Y	Y	Y
13	Bagherpour et al., 2019	Y	U	U	U	U	U	U	Y	Y	Y
14	Ben Halima et al., 2014	Y	U	U	U	U	U	U	Y	Y	Y
15	Ben Slima et al., 2013	Y	U	U	U	U	U	Y	Y	Y	Y
16	Desai et al., 2015	Y	U	U	U	U	U	U	Y	Y	Y
17	El-defrawy et al., 2020	U	U	U	U	U	U	U	Y	Y	Y
18	Osama et al., 2019	Y	U	U	U	U	U	U	Y	Y	Y
19	Radi et al., 2021	Y	U	U	U	U	U	U	Y	Y	Y
20	Sakr and Al-Amoudi 2012	Y	U	U	U	U	U	U	Y	Y	Y
21	Sharma et al., 2016	U	U	U	U	U	U	U	Y	Y	Y
22	Sharma et al., 2018	U	U	U	U	U	U	U	U	U	U
23	Wang et al., 2024	Y	U	U	U	U	U	U	Y	Y	U
24	Jebur et al., 2023a	Y	U	U	U	U	U	U	Y	Y	Y
25	Mohamed et al., 2023	Y	U	U	U	U	U	U	Y	Y	Y
26	Nashed et al., 2024	Y	U	U	U	U	U	U	Y	Y	Y
27	Soliman et al., 2025	Y	U	U	U	U	U	U	Y	Y	Y
28	El-Demerdash et al., 2004	U	U	U	U	U	U	U	Y	Y	U
29	Jebur et al., 2023b	Y	U	U	U	U	U	U	Y	Y	Y
30	Atef et al., 2020	Y	U	U	U	U	U	U	Y	Y	Y
31	Khaki et al., 2017	U	U	U	U	U	U	U	Y	Y	U
32	Rajabzadeh et al., 2018	U	U	U	U	U	U	U	Y	Y	Y
33	Tanushreea et al., 2024	Y	U	U	U	U	U	U	Y	Y	Y

Domains: D1, sequence generation; D2, baseline characteristics; D3, allocation concealment; D4, random housing; D5, blinding of investigators; D6, random outcome assessment; D7, blinding of outcome assessor; D8, incomplete outcome data; D9, selective outcome reporting, and D10, Other sources of bias; Y, Low risk (Green); U, Unclear (Blue); N, High risk (Red).

### 2.5 Reporting quality assessment

The quality of each animal study was assessed using items described in the ARRIVE (animal research: reporting *in vivo* experiments) guidelines (Hooijmans et al., 2011; Luo and Leng, 2013), which are recommended for quality assessment of animal experiments (Supplementary Table S4). The total score was 10 points, and each study was graded as low (0-3 points), medium (4-6 points), or high quality (7-10 points). In addition to assessing reporting quality, the risk of bias of each included study was evaluated using the Systematic Review Centre for Laboratory Animal Experimentation (SYRCLE) (Hooijmans et al., 2014). This tool comprises 10 items across six domains of bias: selection bias (sequence generation, baseline characteristics, and allocation concealment), performance bias (random housing, blinding of caregivers/researchers), detection bias (random outcome assessment, blinding of outcome assessors), attrition bias (incomplete outcome data), reporting bias (selective outcome reporting), and other sources of bias (Supplementary Table S5). Each item was classified as having a low, high, or unclear risk of bias based on the information reported in the original article.

## 3 Results

### 3.1 Study characteristics

All 142 identified articles were reviewed. Figure 1 illustrates the systematic search and selection process. The systematic review included 33 experimental animal studies using male laboratory animals conducted from 2003 to 2025. All studies used male laboratory animals, including rats (*n* = 22), mice (*n* = 6), rabbits (*n* = 3), and guinea pigs (*n* = 2). The studies examined five pyrethroid compounds: cypermethrin (*n* = 12), deltamethrin (*n* = 11), fenpropathrin (*n* = 4), fenvalerate (*n* = 2), and permethrin (*n* = 4) (Table 2).

**FIGURE 1:**
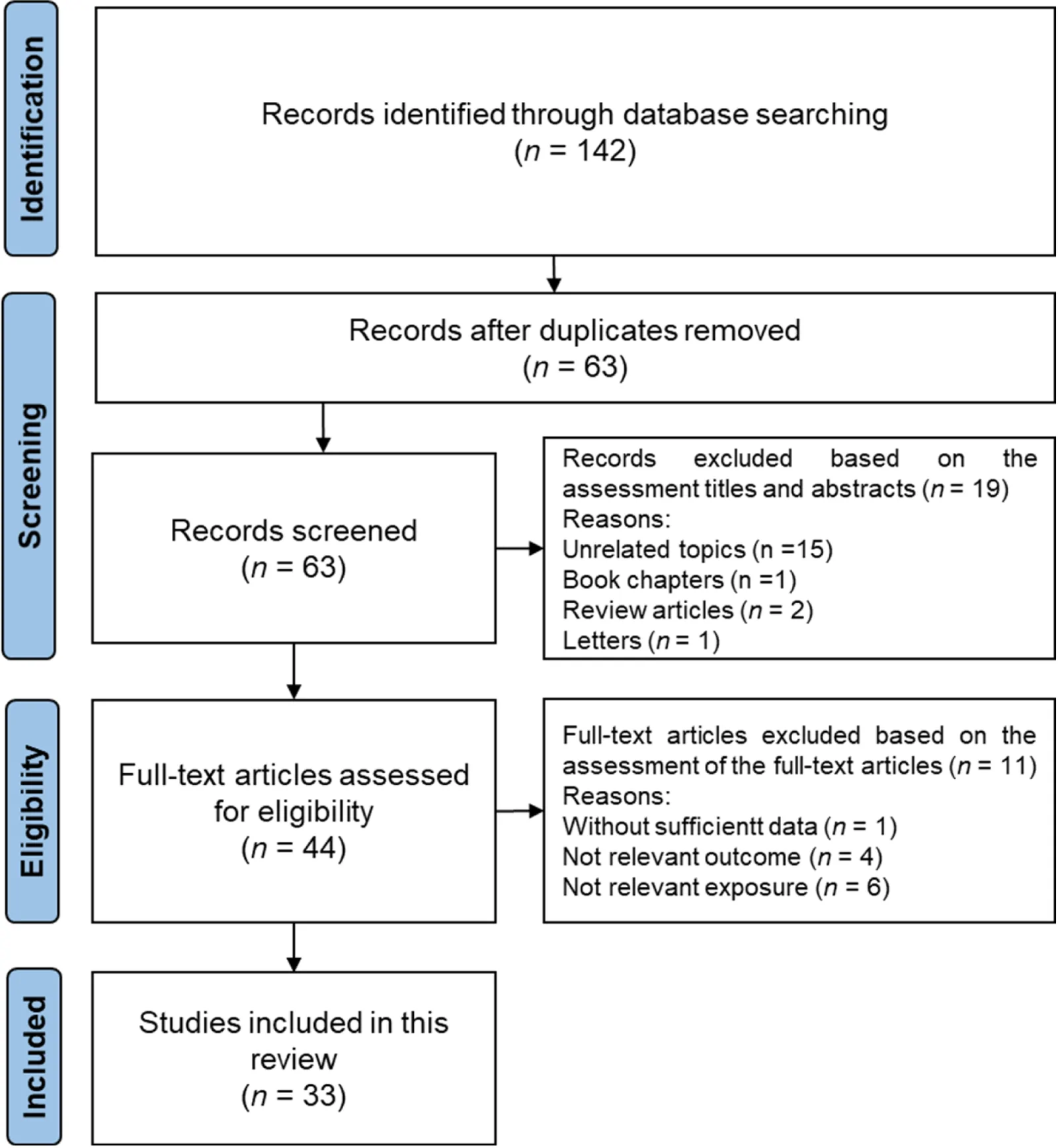
PRISMA diagram of the detailed study selection process.

**TABLE 2:** Description of the animal studies included in the systematic review.

Number	Author, year	Groups	Pyrethroid dose	Natural product(s) and dose	Time of assay	Sample	Method of assessment	Effect of pesticides compared with the control group	Impact of pesticides + natural compounds compared with the insecticide group	Main outcomes
Cypermethrin (CYP)										
1	Abd-Ellaah et al., 2024	32 adult male rats (*n* = 8) 1. Control *2. Panax ginseng* 3. CYP 4. Panax + CYP	9.4 mg/kg	0.1% *Panax* *ginseng*	60 days (5 days weekly)	Sperm,testis and blood	ELISA and H&E	↓ Weight (testis, prostate, seminal vesicle), sperm (count, motility, normality, viability), LH, FSH, and testosterone ↑ Testicular tissue damage	↔ Weight (testis, prostate, seminal vesicle), sperm (count, motility, normality, viability), LH, FSH, and testosterone ↓ Testicular tissue damage	Involvement with CYP caused overt defects in male reproductive function, which were cured by administering the androgenic drug *Panax*
2	Adeniyi et al., 2024	25 male Wistar rats (*n* = 5) 1. Control 2. CYP 3. CYP + Ascorbic acid 4. CYP + α-tocopherol 5. CYP + Ascorbic acid + α-tocopherol	10 mg/kg	Ascorbic acid (5,000 mg/kg) and α-tocopherol (3,000 mg/kg	28 days	Testis	Spectrophotometry and H&E	↑ MDA and testicular tissue damage ↓ SOD, GPx, and CAT.	↓ MDA and testicular tissue damage ↑ SOD, GPx, and CAT.	Coadministration of ascorbic acid and α-tocopherol reduces cypermethrin-induced oxidative stress better than single administration, possibly due to their complementary antioxidant properties
3	Ali Hasan and Al-Rikaby 2023	40 adult male rabbits (*n* = 10) 1. Control 2. CYP 3. Rosemary extract (RE) (100 mg/kg) + CYP 4. RE (200 mg/kg) + CYP	66.5 mg/kg	100 and 200 mg/kg	42 days	Blood, and testis	ELISA and H&E	↓ LH, FSH, estradiol, and testosterone ↑ Testicular tissue damage	↑ LH, FSH, estradiol, and testosterone ↓ Testicular tissue damage	RE possesses anti-infertility and strong antioxidant activities and can be used as a fertility-increasing drug to control sexual hormones and spermatogenesis, preventing toxicity and its pathophysiological consequences
4	Chouabia et al., 2021	30 male Wistar rats (*n* = 5) 1. Control *2. Salvia officinalis* L. extract (SLE) (500 mg) 3. CYP1 (8.33 mg/kg) 4. CYP2 (25 mg/kg) 5. SLE + CYP1 6. SLE + CYP2	8.33 and 25 mg/kg	S*alvia officinalis* L. extract (SLE) (500 mg)	30 days (5 days weekly)	Sperm, and testis	CASA and H&E	↓ Weight (testis and epididymis) and sperm (count and motility) ↑ Sperm velocity and testicular tissue damage	↑ Weight (testis and epididymis), and sperm (count and motility) ↓ Sperm velocity and testicular tissue damage	SLE has been shown to protect rats from CYP-induced reprotoxicity
5	El-Demerdash et al., 2003	24 New Zealand White rabbits (*n* = 6) 1. Control 2. Isoflavone (2 mg/kg) 3. CYP (24 mg/kg) 4. Isoflavone + CYP	24 mg/kg	Isoflavone (2 mg/kg)	84 days	Testis	Spectrophotometry	↑ TBARS. ↓ GST.	↓ TBARS. ↑ GST.	Isoflavone confers marked protection against CYP-induced oxidative stress in rabbits’ testes
6	Herman et al., 2024	24 guinea pigs (*Cavia porcellus*) (*n* = 6) 1. Control 2. CYP (80 mg/kg) 3. CYP + *Stychnos camptoneura* extract (SCE) (100 mg/kg) 4. CYP + SCE (250 mg/kg)	80 mg/kg	SCE (100 and 250 mg/kg)	60 days	Sperm and blood	​	↔ Weight (testis, epididymis, ↓Seminal vesicles, vas deferens, prostate, and penis), Sperm (count, motility, vitality, and mass mobility), ↓ Testosterone	↔ Weight (testis, epididymis, ↑Seminal vesicles, vas deferens, prostate, and penis), Sperm (count, motility, vitality, and mass mobility), ↑ Testosterone	The hydroethanol extract of *Strychnos camptoneura* trunk bark has protective effects against cypermethrin-induced male infertility, due to its androgenic, spermatogenic, and antiradical properties
7	Khormi et al., 2025	24 male Wistar rats (*n* = 6) 1. Control 2. CYP (145 mg/kg) 3. Moringa oil (MO) (60 mL/750 mL of corn oil) 4. CYP + MO	145 mg/kg	MO (60 mL/750 mL of corn oil	28 days	Testis	H&E and molecular docking analysis	↑ Testicular tissue damage ↑ Affinity of Testis-expressed protein 101 (TEX101) to band with CYP	↓ Testicular tissue damage	The molecular docking analysis of a specific testicular protein expressed a high affinity with Cypermethrin active bonds. Moringa oil is considered a dietary supplement that can mitigate the negative effects of artificial ingredients in food
8	Sharma et al., 2013	24 male Wistar rats (*n* = 6) 1. Control 2. CYP (3.38 mg/kg), 3. CYP + *Tribulus terrestris* (EETT) (100 mg/kg) 4. EETT	3.38 mg/kg	EETT (100 mg/kg)	28 days	Sperm, testis and blood	ELISA and spectrophotometry	↓ Weight (testis, epididymis), sperm (count, motility, normality, and viability), testosterone, FSH, LH, GSH, SOD, CAT, GPx, GR, and GST. ↑ LPO.	↑ Weight (testis, epididymis), sperm (count, motility, normality, and viability), testosterone, FSH, LH, GSH, SOD, CAT, GPx, GR, and GST. ↓ LPO.	Cypermethrin-induced alterations in fertility parameters, reproductive hormones, and biochemical parameters were reverted to normal levels by EETT treatment. The EETT plant possesses reproductive system enhancement and antioxidant activity
9	Sharma et al., 2014	30 male Wistar rats (*n* = 6) 1. Control 2. CYP (3.83 mg/kg) 3. CYP (14 days)+ resveratrol (20 mg/kg) (14 days) 4. Resveratrol (14 days) + CYP (14 days) 5. Resveratrol (14 days)	3.83 mg/kg	Resveratrol (20 mg/kg)	28 days	Sperm, and testis	ELISA, and spectrophotometry	↓ Weight (testis, epididymis), sperm (count, motility, normality and viability), LH, FSH, testosterone, CAT, SOD, GPx, GST, GR, and GSH. ↑ LPO.	↔ Weight (testis, epididymis) ↑ Sperm (count, motility, normality and viability), LH, FSH, testosterone, CAT, SOD, GPx, GST, GR, and GSH. ↓ LPO.	The study concluded that resveratrol ameliorated CYP-induced testicular damage by reducing oxidative stress and enhancing sex hormone levels
10	Ubah et al., 2021	42 male Wistar rats (*n* = 6) 1. Control 2. Cypermethrin (CYP) (60 mg/kg) 3. CYP + date fruit extract (250 mg/kg) 4. Date fruit extract (250 mg/kg)	60 mg/kg	Date fruit extract (250 mg/kg)	56 days	Sperm, and blood	RIA kits	↔ Sperm (motility, normality, and viability), mass activity, pH, testosterone, FSH,	↔ Sperm (motility, normality, and viability), mass activity, and pH, ↓ Testosterone, and FSH.	Despite the protective properties of date fruit extracts, the plant also adversely affected male reproductive system fertility, as observed in the present study. The endocrine system was modulated by reductions in testosterone and FSH levels
11	Vemo et al., 2017	50 male guinea pigs (*n* = 10) 1. Control 2. CYP (137.50 mg/kg) 3. CYP + *Bersama engleriana* (BE) (50 mg/kg) 4. CYP + BE (100 mg/kg) 5. CYP + BE (200 mg/kg)	137.50 mg/kg	BE (50, 100 and 200 mg/kg)	90 days	Sperm, blood and testis	ELISA, spectrophotometry and H&E	↓ Testis weight, peroxidase ↔ Weight (epididymis, vas deferens, and prostate), testosterone, and sperm (count, motility, normality) ↑ MDA, SOD, CAT, and testicular tissue damage	↑ Testis weight, peroxidase, testosterone, sperm (count, motility, normality) ↔ Weight (epididymis, vas deferens, and prostate) ↓ MDA, SOD, CAT, and testicular tissue damage	Ethanol extract of *B. engleriana* leaves prevents the induction of oxidative stress and reproductive parameters impairment by CYP in male guinea pigs
12	Yousef et al., 2003	24 male New Zealand White rabbits (*n* = 6) 1. Control 2. Isoflavones (2 mg/kg) 3. CYP (24 mg/kg) 4. Isoflavones + CYP	24 mg/kg	Isoflavones (2 mg/kg)	84 days	Sperm, and blood	RIA kits	↓ Weight (testis and epididymis), sperm (count, motility, normality, and viability), testosterone, ejaculate volume, packed sperm volume, and initial fructose ↑ pH	↑ Weight (testis and epididymis), sperm (count, motility, normality, and viability), testosterone, ejaculate volume, packed sperm volume, initial fructose, and pH	Results showed that isoflavones reduced CYP’s negative effects on the reproductive characteristics of mature male rabbits. Isoflavones alone caused an improvement in some semen quality and had no negative effects on male fertility
Deltamethrin (DEL)										
13	Bagherpour et al., 2019	35 adult male mice (*n* = 7) 1. Control 2. Corn oil (0.2 mL) 3. Green tea extract (GT) (150 mg/kg) DEL (0.6 mg/kg) 4. GT + DEL	0.6 mg/kg	GT (150 mg/kg)	28 days	Sperm, testis and blood	IHC, ELISA, spectrophotometry, and H&E	↓ Sperm (count, motility, normality, and viability), testosterone, Johnsen’s score, epithelial height, and diameter of the seminiferous tubules ↑ MDA and caspase-3 expression	↑ Sperm (count, motility, normality, and viability), testosterone, Johnsen’s score, epithelial height, and diameter of the seminiferous tubules ↓ MDA and caspase-3 expression	The GT treatment, with antioxidant activity and anti-apoptotic properties, could protect against DEL-induced testicular injury
14	Ben Halima et al., 2014	32 Swiss albino male mice (*n* = 8) 1. Control 2. DEL (5 mg/kg) 3. Oat oil (OO) (6 g/kg) 4. DEL + OO	5 mg/kg	OO (6 g/kg)	35 days	Sperm, and testis	Spectrophotometry and H&E	↓ Sperm (count, motility, and normality) ↔ live sperm, GSH, and CAT. ↑SOD, GPx, MDA, and testicular tissue damage	↑ Sperm (count, motility, and normality) ↔ live sperm, SOD, GSH, CAT ↓MDA, GPx, and testicular tissue damage	OO ameliorated the toxic effects of DEL in the test, which was explored
15	Ben Slima et al., 2013	60 male albino mice (*n* = 10) 1. Control 2. DEL (5 mg/kg) 3. DEL + essential oil of *P. graveolens* (EO) (67 mg/kg) 4. DEL + Vit E 5. EO 6. Vit E	5 mg/kg	EO (67 mg/kg)	35 days	Sperm, and testis	Spectrophotometry and H&E	↓ Sperm (count, motility, normality, viability), SOD, GSH, CAT, Ascorbic acid ↑ MDA, PCO, and testicular tissue damage	↑ Sperm (count, motility, normality, viability), SOD, GSH, CAT, Ascorbic acid ↓ MDA, PCO, and testicular tissue damage	EO geranium prevented oxidative damage in the testes, as observed. This study has shown a positive influence of EO on the male reproductive system, similar to that of Vit E
16	Desai et al., 2015	40 male albino mice (*n* = 8) 1. Control 2. Peanut oil 3. DEL (6 mg/kg) *4. Allium sativum* (AS) (200 mg/kg) 5. DEL + AS	6 mg/kg	(AS) (200 mg/kg	45 days	Testis	Spectrophotometry	↓ Testis weight, SOD, CAT, GSH, GPx, GRx, and GST ↑ LPO.	↔ Testis weight, SOD, CAT, GSH, GPx, GRx, and GST LPO.	Results indicate that DEL exerts significant harmful effects on male mice, and the AS group had no significant difference from the DEL group
17	El-defrawy et al., 2020	54 male albino rats (*n* = 6) 1. Control 2. DEL (10 mg/kg) 3. DEL (5 mg/kg) 4. Moringa leaves powder (MLP) (5%) 5. Sage leaves powder (SLP) (2.5%) 6. MLP + DEL (10 mg/kg) 7. MLP + DEL (5 mg/kg) 8. SLP + DEL (10 mg/kg) 9. SLP + DEL (5 mg/kg)	5 and 10 mg/kg	MLP (5%) and SLP (2.5%)	30 days	Sperm, and blood	​	↔ Weight (testis, epididymis, prostate, and seminal vesicle) and testosterone ↓ Sperm (count, motility, and normality)	↔ Weight (testis, epididymis, and prostate) and testosterone ↓ Seminal vesicle weight ↑ Sperm (count, motility, and normality)	Supplementation of daily meals with dried Moringa and sage leaves is recommended to prevent infertility resulting from exposure to DEL.
18	Osama et al., 2019	40 male albino rats (*n* = 10) 1. Control, *2. Chlorella vulgaris* (CV) (50 mg/kg), 3. DEL (3 mg/kg), 4. 4. CV + DEL	3 mg/kg	CV (50 mg/kg)	56 days	Sperm, blood, and testis	ELISA, spectrophotometry and H&E	↓ Weight (testis, epididymis), sperm (count, motility, normality, viability), testosterone, SOD, and CAT. ↑ MDA and testicular tissue damage	↑ Weight (testis, epididymis), sperm (count, motility, normality, viability), testosterone, SOD, and CAT. ↓ MDA and testicular tissue damage	CV administration improved testicular function in normal rats and ameliorated the effects of severe oxidative stress
19	Radi et al., 2021	30 male albino rats (*n* = 10) 1. Control 2. DEL (6 mg/kg) 3. Wheat germ oil (1.5 mL/kg) + DEL	6 mg/kg	Wheat germ oil (1.5 mL/kg)	65 days	Sperm, blood and testis	Enzyme immunoassay, electrophoresis, and H&E	↓ Testis weight, sperm (count, motility, and normality), and testosterone ↑ DNA fragmentation and testicular tissue damage	↑ Testis weight, and sperm (count, motility, and normality) ↔ Testosterone ↓ DNA fragmentation	Wheat germ oil can mitigate the adverse effects of DEL on male fertility and improve male reproductive performance
20	Sakr and Al-Amoudi 2012	70 male Wister rats (*n* = 10) 1. Control 2. Ginger (24 mg/mL) (*n* = 20) 3. DEL (0.6 mg) (*n* = 20) 4. Ginger + DEL (*n* = 20)	0.6 mg	Ginger (24 mg/mL)	42 days (3 days weekly)	Sperm and testis	H&E and IHC	↓ Testis weight, sperm (count, motility), diameter of tubules, germinal epithelial height, and ↑ expression (p53 and bax)	↑ Testis weight, sperm (count, motility), diameter of tubules, germinal epithelial height, and ↓ expression (p53 and bax)	Ginger treatment reduces the expression of p53 and bax. This effect of ginger extract may be attributed to its antioxidant activity
21	Sharma et al., 2016	42 male Wistar rats (*n* = 6) 1. Control 2. DEL (2 mg/kg) *3. Curculigo orchioides* (CO) (100 mg/kg) 4. CO (200 mg/kg) 5. DEL (2 mg/kg) + CO (100 mg/kg) 6. DEL (2 mg/kg) + CO (200 mg/kg) 7. DEL (2 mg/kg) allowed to metabolize for 1 month	2 mg/kg	CO (100 and 200 mg/kg (	60 days	Sperm, testis, and blood	ELISA, spectrophotometry and H&E	↓ Weight (testis, prostate, seminal vesicle, cauda epididymis), sperm (count, motility, normality), testosterone, FSH, LH, GSH, SOD, CAT, GPx, GR, GST, 3β-HSD, and 17β HSD. ↑ LPO and testicular tissue damage	↑ Weight (testis, prostate, seminal vesicle, cauda epididymis), sperm (count, motility, normality), testosterone, FSH, LH, GSH, SOD, CAT, GPx, GR, GST, 3β HSD, and 17β HSD. ↓ LPO and testicular tissue damage	The findings indicated that DLM damaged the reproductive system of male Wistar rats. CO treatment efficiently improves the reproductive system, chiefly by increasing levels of important steroidogenic enzymes and hormones
22	Sharma et al., 2018	42 male Wistar rats (*n* = 6) 1. Control 2. CYP (2 mg/kg.bw) 3. DEL (2 mg/kg.bw) 4. CYP + DEL 5. CYP + DEL + curcumin (CUR) (100 mg/kg.bw) 6. CYP + DEL + quercetin (100 mg/kg.bw) 7. CYP + DEL + curcumin (CUR) + quercetin	CYP and DEL (2 mg/kg.bw)	CUR (100 mg/kg.bw)and Qquercetin (100 mg/kg.bw)	45 days	Sperm, and testis and blood	ELISA spectrophotometry and H&E	↓ Weight (testis, epididymis), sperm (count, motility, normality), LH, FSH, testosterone, 3β-HSD, 17-β HSD, CAT, SOD, GPx, GST, GR, and GSH, ↑ LPO and testicular tissue damage	↑ Weight (testis, epididymis), sperm (count, motility, normality), LH, FSH, testosterone, 3-β HSD, 17-β HSD, CAT, SOD, GPx, GST, GR, and GSH, ↓ LPO and testicular tissue damage	Curcumin and quercetin protected against CYP and DEL-induced reproductive system toxicity and oxidative damage in rats. The increases in the activities of 3β-HSD and 17β-HSD, with concomitant increases in testosterone, were primarily responsible for the ameliorative effects of curcumin and quercetin. Curcumin showed slightly greater activity than quercetin. The combination of the two antioxidants offered greater protection than either alone
23	Wang et al., 2024	120 ICR male mice (*n* = 30) 1. Control 2. DEL (10 mg/kg) 3. DEL + *Ginkgo biloba* extract (GBE) (100 mg/kg) 4. GBE (100 mg/kg)	10 mg/kg	GBE (100 mg/kg)	30 days	Blood and testise	ELISA, H&E, periodate-schiff (PAS), immunohistochemistry, immunofluorescence, RT-qPCR, Western blotting, Cell culture	↓ Testis index, Anogenital distance, INH-B, Testosterone, number of Spermatids and sertoli cells, Testicular Johnsen’s score ↓ relative mRNA levels (TNP1, TNP2, PRM1, and PRM2) Protein expression (↑H2B, H3, and ↓ TNP2)	↑ Testis index, Anogenital distance, INH-B, Testosterone, number of spermations and sertoli cells, Testicular Johnsen’s score ↑ relative mRNA levels (TNP1, TNP2, PRM1, and PRM2) Protein expression (↓ H2B, H3, and ↑TNP2)	GBE has demonstrated notable efficacy in alleviating the reproductive toxicity induced by DEL. It exerts therapeutic effects by ameliorating autophagy and reducing DEL-induced testicular damage
Fenpropathrin (FNP)										
24	Jebur et al., 2023a	28 male Wistar rats (*n* = 7) 1. Control *2. P. granatum* peel extract (PGPE) (500 mg/kg) 3. Fenpropathrin (FNP) (15 mg/kg) 4. PGPE + FNP	15 mg/kg	PGPE (500 mg/kg)	28 days	Sperm, testis and blood	RIA, spectrophotometry, RT-PCR, ELISA and H&E	↓ Testis weight, gonadosomatic index, sperm (count, motility, normality, viability), testosterone, SOD, CAT, GPx, GR, GST, GSH, and expression (Bcl2 and IL-10) ↑ FSH, LH, TBARS, H_2_O_2_, PCC, and expression (P53, Caspase-3 and IL-1β)	↑ Testis weight, gonadosomatic index, sperm (count, motility, normality, viability), testosterone, SOD, CAT, GPx, GR, GST, GSH, and expression (Bcl2, IL-10) ↓ FSH, LH, TBARS, H_2_O_2_, PCC and expression (P53, Caspase-3, and IL-1β)	PGPE-pretreated FNP-intoxicated rats showed considerable improvement in most studied parameters compared with FNP-treated groups. Conclusively, PGPE provided a potent protective effect against the testicular toxicity caused by FNP, due to its antioxidant-active components
25	Mohamed et al., 2023	60 male SD rats (*n* = 10) 1. Control, 2. Curcumin (CUR) (50 mg/kg), 3. Chitosan-encapsulated curcumin nanoparticles (Cs.CUR.NPs.) (50 mg/kg), 4. FNP (15 mg/kg), 5. FNP + CUR, 6. FNP + Cs.CUR.NPs	15 mg/kg	7. CUR (50 mg/kg), and Cs.CUR.NPs (50 mg/kg)	60 days	Sperm, blood and testis	ELISA, H&E and RT-PCR	↓ Testes weight, gonadosomatic index, sperm (count, motility, normality), testosterone, FSH, LH, GPX, GSSG/GSH, GSH, SOD, and expression (StAR, CYP11A1, CYP17A1, CYP19A1, HSD17B3, Kiss-1, Kiss-r1, and GLP-1). ↑ estradiol and MDA.	↑ Testes weight, gonadosomatic index, sperm (count, motility, normality), testosterone, FSH, LH, GPX, GSSG/GSH, GSH, SOD, and expression (StAR, CYP11A1, CYP17A1, CYP19A1, HSD17B3, Kiss-1, Kiss-r1, and GLP-1) ↓ estradiol and MDA.	The treatment with CUR and Cs.CUR.NPs exhibited remarkable restorative effects on semen quality, sex hormone levels, antioxidant capacity, and mRNA expression of the targeted genes. Notably, Cs.CUR. NPs exhibited superior properties compared with CUR.
26	Nashed et al., 2024	30 male Wistar rats (*n* = 5) 1. Control *2. Sambucus nigra* (SN) (200 mg/kg) 3. SN (400 mg/kg) 4. FNP (4.7 mg/kg) 5. FNP + SN (200 mg/kg) 6. FNP + SN (400 mg/kg)	4.7 mg/kg	SN (200 and 400 mg/kg)	60 days	Sperm, and testis and blood	IHC, RT-PCR, ELISA, spectrophotometry, and H&E	↓ Sperm (count, motility), testosterone, expression (StAR, caspase 3), GSH, leydig cell counts, diameter of seminiferous tubules, and thickness of germ cell layers ↑ MDA, area of total interstitial Space	↑ Sperm (count, motility), testosterone, expression (StAR, caspase 3), GSH, Johnsen score, leydig cell counts, diameter of seminiferous tubules, and thickness of germ cell layers ↓ MDA, area of total interstitial Space	The data indicate that FNP can induce testicular harm and infertility, but SN extract can mitigate these detrimental consequences
27	Soliman et al., 2025	30 male Wistar rats (*n* = 5) 1. Control 2. Date palm kernel extract (DPK) (200 mg/kg) 3. DPK (400 mg/kg) 4. FNP (4.7 mg/kg) 5. FNP + DPK (200 mg/kg) 6. FNP + DPK (400 mg/kg)	4.7 mg/kg	DPK (200 and 400 mg/kg)	60 days	Sperm, and testis and blood	IHC, RT-PCR, ELISA, spectrophotometry, and H&E	↓ Sperm (count, motility), testosterone, expression (StAR, caspase-3), GSH, leydig cell counts, diameter of seminiferous tubules, and thickness of germ cell layers ↑ MDA, desquamation, and interstitial space edema	↑ Sperm (count, motility), testosterone, expression (StAR, caspase-3), GSH, leydig cell counts, diameter of seminiferous tubules, and thickness of germ cell layers ↓ MDA, desquamation, and Interstitial space edema	FNP caused oxidative stress, reduced sperm count, and impaired motility. FNP reduced StAR gene expression and lowered serum testosterone levels. When taken orally, DPK extract may protect against FNP-induced male reproductive toxicity
Fenvalerate (FEN)										
28	El-Demerdash et al., 2004	56 male rats (*n* = 7) 1. Control 2. β-carotene (10 mg/kg) 3. Vitamin E (100 mg/kg) 4. β-carotene + Vit. E 5. FEN (20 mg/kg) 6. FEN + β-carotene 7. FEN + Vit E 8. FEN + β-carotene + Vit E	20 mg/kg	β-carotene (10 mg/kg) and Vit E (100 mg/kg)	30 days	Blood and sperm	Spectrophotometry	↔ TBARS, GST, and sperm count ↓Sperm (motility, normality, viability),	↓ TBARS, GST, ↔ Sperm count ↑Sperm (motility, normality, viability)	β-carotene improved semen quality and minimized FEN’s toxic effects
29	Jebur et al., 2023b	28 male Wistar rats (*n* = 7) 1. Control 2. Lycopene (LYC) (10 mg/kg) 3. FEN (20 mg/kg) 4. LYC + FEN	20 mg/kg	LYC (10 mg/kg)	30 days	Blood, testis and sperm	RIA kits, and qRT-PCR	↓ Sperm (count, motility, normality, viability), testosterone, CAT, SOD, GPx, GST, GR, GSH, and ↑LH, FSH, TBARS, H_2_O_2_, and testicular tissue damage and expression (caspase-8, caspase-3)	↑ Sperm (count, motility, normality, viability), testosterone, CAT, SOD, GPx, GST, GR, GSH, and ↓ LH, FSH, TBARS, H_2_O_2_, and testicular tissue damage and expression (caspase-8, caspase-3)	Lycopene may have antioxidant and preventive properties against FEN-induced testicular damage
Permethrin (PER)										
30	Atef et al., 2020	30 male Wistar rats (*n* = 10) 1. Control 2. PER (35 mg/kg) 3. PER + matrine (100 mg/kg)	35 mg/kg	Matrine (100 mg/kg)	28 days	Sperm, testis and blood	ELISA, spectrophotometry, DPACA, H&E and RT-PCR	↓ Weight (testes, epididymis), sperm (count, normality), testosterone, GPx, TAC, and testicular StAR. ↑ MDA, % fragmented DNA, testicular p-ERK1/2, testicular COX-2, and testicular tissue damage	↑ Weight (testes, epididymis), sperm (count, normality), testosterone, GPx, TAC, and testicular StAR. ↓ MDA, % fragmented DNA, testicular p-ERK1/2, testicular COX-2, and testicular tissue damage	Matrine can be used as a promising therapeutic agent in the treatment of PER toxicity
31	Khaki et al., 2017	30 male Wister rats (*n* = 6) 1. Control 2. DMSO as the solution (0/5 mL) 3. PER (35 mg/kg) 4. PER +3 mL of onion juice 5. Onion juice (3 mL)	35 mg/kg	Onion juice (3 mL)	60 days	Sperm, leydig cell, and blood	Double-antibody immunoassay	↓ Weight (testes, prostate, seminal vesicle), sperm (motility, normality, viability), leydig cell counts, and testosterone	↑ Weight (testes, prostate, seminal vesicle), sperm (motility, normality, viability), leydig cell counts, and testosterone	Permethrin has negative effects on the reproductive system and fertility, and onion showed a significant remedial effect on sperm parameters, and leads to an increase in the number of leydig cells
32	Rajabzadeh et al., 2018	30 male Wistar rats (*n* = 6) 1. Control 2. DMSO (0.5 mL) 3. PER (35 mg/kg) 4. PER +3 mL of onion juice Onion juice	35 mg/kg	Onion juice (3 mL)	60 days	Testis and blood	Real time-PCR, and double-antibody immunoassay,	↓ expression (SF1, LHCGR), FSH, and LH.	↑ expression (SF1, LHCGR) ↔ FSH and LH.	The toxicity effects of PER can reduce male fertility. Therefore, consuming natural antioxidants like onions can reduce damage
33	Tanushreea et al., 2024	15 adult male mice (*n* = 5) 1. Control 2. PER (130 mg/kg) 3. PER + fenugreek aqueous extract (100 mg/kg)	130 mg/kg	Fenugreek aqueous extract (100 mg/kg)	20 days	Sperm and testis	​	↓ Weight (testes, seminal vesicle, vas deferens) and sperm count	↑ Weight (testes, seminal vesicle, vas deferens) and sperm count	Fenugreek aqueous extract may mitigate pesticide-induced gonadotoxicity in the reproductive organs of male Swiss albino mice

Abbreviations: AS: allium sativum, BAX: Bcl-2-associated X protein, Bcl2: B-cell lymphoma 2, BE: bersama engleriana, CASA: computer-assisted semen analysis, CAT: catalase, COX-2: cyclooxygenase 2, Cs.CUR.NPs.: chitosan-encapsulated curcumin nanoparticles, CUR: curcumin, CV: chlorella vulgaris, CYP: cypermethrin, CYP11A1: cytochrome P450 11A1, CYP17A1: cytochrome P450 17A1, CYP19A1: aromatase, DEL: deltamethrin, DMSO: dimethyl sulfoxide, DPACA: diphenylamine colorimetric assay, DPKD: date palm kernel extract, ELISA: enzyme-linked immunosorbent assay, FEN: fenvalerate, FNP: fenpropathrin, FSH: follicle-stimulating hormone, GBE: ginkgo biloba extract, GLP-1: glucagon-like peptide-1, GPx: glutathione peroxidase, GR: glutathione reductase, GSH: glutathione, GST: glutathione S-transferase, GT: green tea extract, H_2_O_2_: hydrogen peroxide, H&E: hematoxylin and eosin, H2B, histone H2B, H3: histone H3, HSD17B3: 17β-hydroxysteroid dehydrogenase type 3, IHC: immunohistochemical staining; IL-10: interleukin-1β, INHB: inhibin B, Kiss-1: kisspeptin, Kiss-r1: kisspeptin receptor, LH: luteinizing hormone, LHCGR: luteinizing hormone/choriogonadotropin receptor, LYC: lycopene, MLP: moringa leaves powder, MO: moringa oil, OO: oat oil, P53: tumor protein p53, PAS, periodate-schiff, p-ERK1/2: phosphorylated extracellular signal-regulated kinase ½. PCC: protein carbonyl content, PER: permethrin, PGPE: punica granatum peel extract, PRM1: protamine 1, PRM2: protamine 2, qRT-PCR: quantitative reverse transcription polymerase chain reaction, RE: rosemary extract, RIA: radioimmunoassay, RT-PCR: real-time polymerase chain reaction; SCE: stychnos camptoneura extract, SF-1: steroidogenic factor 1, SLE: salvia officinalis extract, SLP: sage leaves powder, SN: sambucus nigra, SOD: superoxide dismutase, StAR: steroidogenic acute regulatory protein, TBARS: thiobarbituric acid-reactive substances, TEX101: testis-expressed protein 101, TNP1: transition nuclear protein 1, TNP2: transition nuclear proteins 2, EETT: ethanolic extract Tribulus terrestris. Use the symbols ↑: significant increase, ↓: significant decrease, ↔ no significant change, to compare pesticide versus control or natural product + pesticide versus pesticide.

### 3.2 Effects of pyrethroids on reproductive parameters

All pyrethroids consistently demonstrated significant negative effects on sperm parameters, including a decrease in sperm count, viability, motility, and normality (Yousef et al., 2003; El-Demerdash et al., 2004; Sakr and Al-Amoudi, 2012; Sharma et al., 2013; Sharma et al., 2013; 2014; 2016; 2018; Ben Slima et al., 2013; Ben Halima et al., 2014; Narcisse et al., 2017; Khaki et al., 2017; Osama et al., 2019; Bagherpour et al., 2019; Atef et al., 2020; El-Defrawy et al., 2020; Radi et al., 2021; Chouabia et al., 2021; Mohamed et al., 2023; Jebur A. B. et al., 2023; Abd-Ellaah et al., 2024; Nashed et al., 2024). Reproductive hormone responses were not completely consistent across studies. Several studies reported reductions in testosterone, LH, and/or FSH following pyrethroid exposure, whereas other studies reported increases or no statistically significant changes in individual gonadotropins. This variability was observed across different pyrethroids, animal species, doses, exposure durations, and experimental designs. Therefore, the available evidence does not support a uniform direction of change for LH and FSH across all experimental models (Sharlip et al., 2002; Yousef et al., 2003; Sharma et al., 2013; Sharma et al., 2014; 2016; Narcisse et al., 2017; Rajabzadeh et al., 2018; Osama et al., 2019; Atef et al., 2020; Ali Hasan and Al-Rikaby, 2023; Mohamed et al., 2023; Abd-Ellaah et al., 2024; Nashed et al., 2024).

Pyrethroid exposure consistently increased oxidative stress, evidenced by elevated malondialdehyde (MDA) and lipid peroxidation (LPO), decreased antioxidant enzyme activities (SOD, CAT, GPx, GST, and GR), and reduced GSH levels (Ben Slima et al., 2013; Sharma et al., 2013; Sharma et al., 2014; Desai et al., 2015; Sharma et al., 2016; 2018; Bagherpour et al., 2019; Osama et al., 2019; Atef et al., 2020; Jebur et al., 2023b; Jebur et al., 2023a.; Mohamed et al., 2023; Nashed et al., 2024).

Studies reporting histological examination showed significant testicular tissue damage, including disrupted seminiferous tubule structure, reduced germinal epithelial height, Leydig cell counts, and increased interstitial space (Ben Slima et al., 2013; Ben Halima et al., 2014; Sharma et al., 2016; 2018; Narcisse et al., 2017; Osama et al., 2019; Atef et al., 2020; Chouabia et al., 2021; Radi et al., 2021; Ali Hasan and Al-Rikaby, 2023; Jebur A. et al., 2023; Abd-Ellaah et al., 2024).

### 3.3 Effects of natural products on reproductive organ weight and sperm parameters

For cypermethrin, several natural products significantly improved organ weight and/or sperm parameters relative to the cypermethrin-only groups. *Salvia officinalis* L. [Lamiaceae] extract increased testis and epididymis weight and sperm count and motility, while reducing the cypermethrin-induced increase in sperm velocity (Chouabia et al., 2021). *Strychnos camptoneura* Gilg & Busse [Loganiaceae] extract (100 and 250 mg/kg) increased seminal vesicle, vas deferens, prostate, and penis weight, sperm count, motility, vitality, and mass mobility, and testosterone, without significantly changing testis or epididymis weight (Herman et al., 2024). Ethanolic extract of *Tribulus terrestris* L. [Zygophyllaceae] (EETT) increased testis and epididymis weight, sperm count, motility, normality, and viability, together with testosterone, FSH, and LH (Sharma, P. et al., 2013). *Bersama engleriana* Gürke [Francoaceae] leaf extract increased testis weight, sperm count, motility, and normality, and testosterone, without significantly changing epididymis, vas deferens, or prostate weight (Vemo et al., 2017), and isoflavones (2 mg/kg) increased testis and epididymis weight, sperm count, motility, normality, and viability, testosterone, ejaculate volume, packed sperm volume, and initial fructose concentration in cypermethrin-exposed rabbits (YousefEl-Demerdash and Al-Salhen, 2003). By contrast, *Panax ginseng* C.A.Mey. [Araliaceae] did not significantly change testis, prostate, or seminal vesicle weight, sperm count, motility, normality, or viability, LH, FSH, or testosterone relative to the cypermethrin-only group, and only testicular tissue damage was significantly reduced (Abd-Ellaah et al., 2024), similarly, resveratrol did not significantly change testis or epididymis weight, although it increased sperm count, motility, normality, and viability together with LH, FSH, and testosterone (Sharma et al., 2014).

For deltamethrin, green tea extract increased sperm count, motility, normality, and viability together with testosterone (Bagherpour et al., 2019); *Chlorella vulgaris* Beijerinck [Chlorellaceae] increased testis and epididymis weight, sperm count, motility, normality, and viability, and testosterone (Osama et al., 2019); wheat germ oil increased testis weight and sperm count, motility, and normality (Radi M. et al., 2021); *Curculigo orchioides* Gaertn. [Hypoxidaceae] (100 and 200 mg/kg) increased testis, prostate, seminal vesicle, and cauda epididymis weight together with sperm count, motility, and normality, testosterone, FSH, and LH (Sharma, P., et al., 2016), ginger extract increased testis weight and sperm count and motility (Sakr and Al-Amoudi, 2012), *Ginkgo biloba* L. [Ginkgoaceae] extract increased testis index, anogenital distance, inhibin B, testosterone, and the number of spermatids and Sertoli cells (Wang et al., 2024), and curcumin and quercetin increased testis and epididymis weight, sperm count, motility, and normality, and testosterone, LH, and FSH, in rats co-exposed to cypermethrin and deltamethrin, with curcumin showing slightly greater activity than quercetin and the combination of both compounds providing greater protection than either alone (Sharma, P., et al., 2018). Oat oil and *Pelargonium graveolens* L'Hér. [Geraniaceae] essential oil each increased sperm count, motility, and normality; oat oil did not significantly change live sperm count relative to the deltamethrin-only group (Ben Halima et al., 2014; Ben Slima et al., 2013). Moringa and sage leaf powder increased sperm count, motility, and normality without significantly changing testis, epididymis, or prostate weight or testosterone, although seminal vesicle weight was further reduced relative to the deltamethrin-only group (El-Defrawy et al., 2020).

For fenpropathrin, *Punica granatum* L. [Lythraceae] peel extract increased testis weight, gonadosomatic index, sperm count, motility, normality, and viability, and testosterone (Jebur A. B. et al., 2023), curcumin and chitosan-encapsulated curcumin nanoparticles increased testes weight, gonadosomatic index, sperm count, motility, and normality, testosterone, FSH, and LH, with the nanoparticle formulation reported to exert greater effects than conventional curcumin (Mohamed et al., 2023) and *Sambucus nigra* L. [Viburnaceae] extract and date palm kernel extract each increased sperm count, motility, and testosterone (Nashed et al., 2024; Soliman et al., 2025). For fenvalerate, β-carotene increased sperm motility, normality, and viability without significantly changing sperm count (El-Demerdash et al., 2004), and lycopene increased sperm count, motility, normality, viability, and testosterone (Jebur et al., 2023a). For permethrin, matrine increased testes and epididymis weight and sperm count, normality, and testosterone (Atef et al., 2020); onion juice increased testes, prostate, and seminal vesicle weight, sperm motility, normality, and viability, Leydig cell counts, and testosterone (Khaki et al., 2017), and fenugreek aqueous extract increased testes, seminal vesicle, and vas deferens weight together with sperm count (Tanushreea and Kumara, 2024).

### 3.4 Effects of natural products on oxidative stress and antioxidant biomarkers

Across pesticide groups, natural products consistently decreased pyrethroid-induced increases in MDA, LPO, thiobarbituric acid-reactive substances (TBARS), and protein carbonyl content (PCC), while increasing SOD, CAT, GPx, GR, GST, and reduced GSH, relative to the corresponding pesticide-only groups. This pattern was reported for ascorbic acid and α-tocopherol against cypermethrin (Adeniyi, T. et al., 2023a), isoflavones against cypermethrin (El-DemerdashYousef and Al-Salhen, 2003), EETT and resveratrol against cypermethrin (Sharma et al., 2013; Sharma et al., 2014), curcumin and quercetin against combined cypermethrin and deltamethrin exposure, which also decreased testicular tissue damage (Sharma et al., 2018), *B. engleriana* extract against cypermethrin (Vemo et al., 2017), green tea extract, *P. graveolens* essential oil, *C. vulgaris*, and *C. orchioides* against deltamethrin (Bagherpour et al., 2019; Ben Slima et al., 2013; Osama et al., 2019; Sharma, P., et al., 2016), *P. granatum* peel extract and curcumin/curcumin nanoparticles against fenpropathrin (Jebur A. et al., 2023; Mohamed et al., 2023), lycopene against fenvalerate (Jebur et al., 2023a), and matrine against permethrin, which additionally increased total antioxidant capacity (Atef et al., 2020), Oat oil decreased MDA and GPx but did not significantly change SOD, GSH, or CAT relative to the deltamethrin-only group (Ben Halima et al., 2014), and β-carotene decreased TBARS and GST activity without significantly changing sperm count relative to the fenvalerate-only group (El-Demerdash et al., 2004). By contrast, *Allium sativum* L. [Amaryllidaceae] (200 mg/kg) produced no significant difference from the deltamethrin-only group in testis weight, SOD, CAT, GSH, GPx, GR, GST, or LPO (Desai et al., 2015), and date fruit extract did not significantly change sperm motility, normality, viability, mass activity, or pH relative to the cypermethrin-only group, while testosterone and FSH were further reduced (Ubah et al., 2021).

### 3.5 Histopathological findings

Histopathological assessment showed that most natural products significantly reduced pyrethroid-induced testicular tissue damage, and several also restored specific structural features of the seminiferous epithelium relative to the pesticide-only groups. Reductions in testicular tissue damage were reported for *P. ginseng*, *S. officinalis* extract, rosemary extract, EETT, and *B. engleriana* extract against cypermethrin (Sharma, P. et al., 2013; Vemo et al., 2017; Abd-Ellaah et al., 2024; Ali Hasan and Al-Rikaby, 2023; Chouabia et al., 2021), Moringa oil against cypermethrin (Khormi et al., 2025), green tea extract, oat oil, *P. graveolens* essential oil, *C. vulgaris*, and *C. orchioides* against deltamethrin (Bagherpour et al., 2019; Ben Halima et al., 2014; Ben Slima et al., 2013; Osama et al., 2019; Sharma, P., et al., 2016), curcumin and quercetin against combined cypermethrin and deltamethrin exposure (Sharma et al., 2018), and matrine against permethrin (Atef et al., 2020). Beyond overall tissue-damage scores, green tea extract increased Johnsen’s score, germinal epithelial height, and seminiferous tubule diameter that had been reduced by deltamethrin (Bagherpour et al., 2019), and ginger extract increased seminiferous tubule diameter and germinal epithelial height in deltamethrin-exposed rats (Sakr and Al-Amoudi, 2012), *S. nigra* extract and date palm kernel extract each increased Leydig cell counts, seminiferous tubule diameter, and germ cell layer thickness, and reduced the pesticide-induced increase in interstitial space area, in fenpropathrin-exposed rats; date palm kernel extract additionally reduced epithelial desquamation and interstitial oedema (Nashed et al., 2024; Soliman et al., 2025). Onion juice increased Leydig cell counts in permethrin-exposed rats (Khaki et al., 2017).

### 3.6 Molecular and gene/protein expression findings

*Curculigo orchioides* increased testicular 3β-hydroxysteroid dehydrogenase (3β-HSD) and 17β-hydroxysteroid dehydrogenase (17β-HSD) activity in deltamethrin-exposed rats (Sharma et al., 2016), and curcumin and quercetin similarly increased 3β-HSD and 17β-HSD activity and testosterone in rats co-exposed to cypermethrin and deltamethrin; curcumin was slightly more effective than quercetin, and the combination provided greater protection than either compound alone (Sharma et al., 2018). Curcumin and chitosan-encapsulated curcumin nanoparticles increased testicular expression of StAR, CYP11A1, CYP17A1, CYP19A1, HSD17B3, Kiss-1, Kiss1r, and GLP-1, and decreased estradiol and MDA in fenpropathrin-exposed rats, with the nanoparticle formulation reported to exert greater effects than free curcumin (Mohamed et al., 2023). Lycopene decreased testicular caspase-8 and caspase-3 expression, which increased with fenvalerate (Jebur et al., 2023a). *Punica granatum* peel extract increased Bcl-2 and IL-10 expression and decreased p53, caspase-3, and IL-1β expression in fenpropathrin-exposed rats (Jebur et al., 2023), and *S. nigra* extract and date palm kernel extract each increased testicular StAR expression in fenpropathrin-exposed rats (Nashed et al., 2024; Soliman et al., 2025). *Ginkgo biloba* extract increased mRNA levels of TNP1, TNP2, PRM1, and PRM2, increased TNP2 protein expression, and decreased H2B and H3 protein expression, reversing the shift induced by deltamethrin (Wang et al., 2024). Matrine increased testicular StAR expression and decreased p-ERK1/2 and COX-2 expression, together with reduced DNA fragmentation, in permethrin-exposed rats (Atef et al., 2020), and onion juice increased expression of SF1 and LHCGR in permethrin-exposed rats (Rajabzadeh and Khaki 2018). A complementary molecular docking analysis indicated high-affinity binding between cypermethrin and testis-expressed protein 101 (TEX101), in the same study in which Moringa oil reduced cypermethrin-induced testicular tissue damage (Khormi et al., 2025).

### 3.7 Immunohistochemical findings

Some studies have used immunohistochemistry to confirm molecular- and cellular-level changes. Green tea extract reduced caspase-3 immunostaining in deltamethrin-exposed testicular tissue (Bagherpour et al., 2019), and ginger extract reduced p53 and Bax immunoexpression in deltamethrin-exposed rats (Sakr and Al-Amoudi, 2012). *Sambucus nigra* extract and date palm kernel extract were each evaluated by immunohistochemistry for StAR and caspase-3 expression in fenpropathrin-exposed rats, alongside the histological changes described above (Nashed et al., 2024; Soliman et al., 2025).

### 3.8 Exceptions and negative findings

The efficacy of natural products was not uniform across the included studies, and several exceptions to the general pattern of protection were noted directly in Table 2. *Allium sativum* (200 mg/kg) showed no significant difference from the deltamethrin-only group in testis weight or in oxidative stress markers (SOD, CAT, GSH, GPx, GR, GST, LPO) (Desai et al., 2015). Date fruit extract did not significantly change sperm motility, normality, viability, mass activity, or pH relative to the cypermethrin-only group, and testosterone and FSH were further reduced (Ubah et al., 2021). Moringa and sage leaf powder further reduced seminal vesicle weight relative to the deltamethrin-only group despite improving sperm count, motility, and normality (El-Defrawy et al., 2020). *Panax ginseng* and resveratrol did not significantly change testis or epididymis weight relative to the cypermethrin-only group, although other sperm, hormonal, and/or antioxidant parameters improved (Abd-Ellaah et al., 2024), and onion juice increased SF1 and LHCGR expression in permethrin-exposed rats without significantly changing FSH or LH (Rajabzadeh and Khaki 2018).

### 3.9 Quality assessment

The final column of Supplementary Table S1 presents the quality assessment results. Most studies scored between 8 and 10 on methodological quality assessment, indicating moderate to high rigor in experimental design, housing conditions, and statistical analysis.

The internal validity (risk of bias) of the 33 included studies was evaluated using SYRCLE’s Risk of Bias tool (Table 1).

Across the six bias domains, random sequence generation was explicitly reported (low risk) in 21/33 studies (64%), while baseline comparability, allocation concealment, random housing, blinding of caregivers/investigators, and random outcome assessment were unclear (i.e., not reported) in all 33 studies (100%). Blinding of the outcome assessor was reported in only 1/33 studies (3%). Incomplete outcome data and selective outcome reporting were both judged low risk in 32/33 studies (97%), and other sources of bias (e.g., conflict-of-interest or ethics-approval statements) were low risk in 26/33 studies (79%). This pattern (Figure 2) indicates that, while attrition and outcome reporting were generally well handled, the included literature rarely reports procedural safeguards (allocation concealment, blinding, and randomized housing).

**FIGURE 2:**
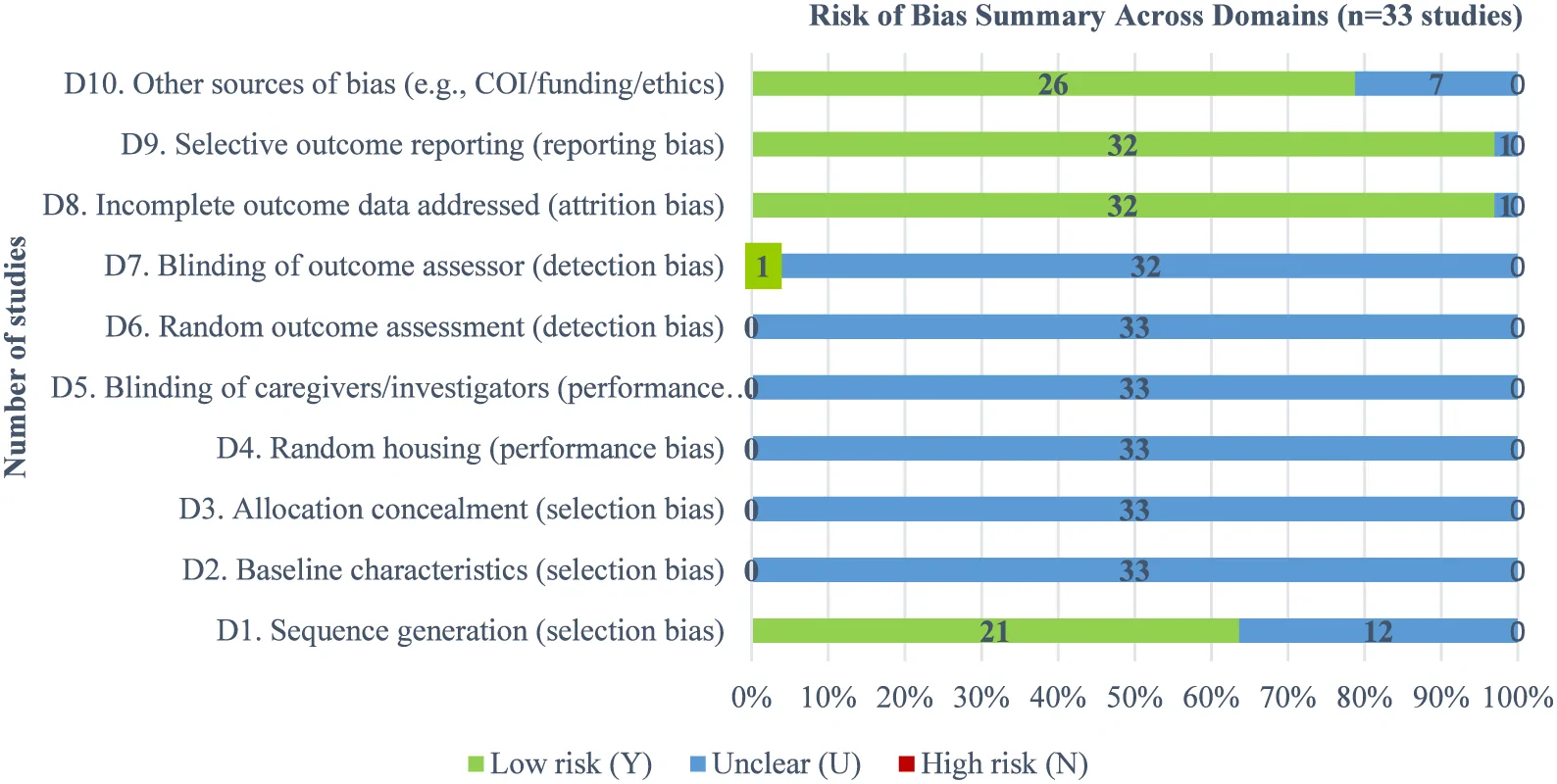
Risk of bias summary for the 33 included studies, assessed using SYRCLE’s Risk of Bias tool for animal studies. Bars show the percentage of studies rated as low risk, unclear risk, or high risk for each of the 10 items across six bias domains (D1-D10).

## 4 Discussion

### 4.1 Overview of findings

This systematic review investigates the evidence on natural products that may reduce pyrethroid-induced reproductive toxicity in male experimental animals. A recently published narrative review has comprehensively discussed the testicular toxicity of various insecticides and the potential of natural products (Darwish et al., 2025). This systematic review complements and extends these findings by providing a focused, structured, and quantitative synthesis of evidence specifically for the pyrethroid class, using a predefined PICO framework and standardized assessment of study quality. Our research shows that pyrethroid exposure frequently results in testicular damage while triggering oxidative stress, hormonal imbalance, and causing severe deterioration of sperm characteristics, including reduced sperm count, lowered motility, and viability. Our review, however, shows that multiple natural substances, including antioxidants, bioactive compounds, plant extracts, and essential oils, protect against pyrethroid-induced reproductive damage.

### 4.2 Reproductive toxicity caused by pyrethroids: an increasing concern

The main cause of pyrethroid toxicity is the excessive production of reactive oxygen species (ROS) that overwhelm antioxidant defense systems, leading to oxidative stress (Kale et al., 1999). Reduced activity of antioxidant enzymes such as CAT and SOD exacerbates the negative effects of pyrethroid exposure (Sharma et al., 2013; Adeniyi et al., 2024).

### 4.3 Protective effects of natural products: antioxidants at the forefront

The reviewed evidence suggests that several natural substances are promising candidates for mitigating the adverse effects of pyrethroid exposure. The protective properties of sage (*S. officinalis*) among various antioxidant-rich plant extracts have proved effective against cypermethrin-induced reproductive toxicity. When co-treated with cypermethrin, the aqueous extract of sage enhanced sperm parameters and reproductive organ weights, demonstrating its ability to counteract the toxic effects of pyrethroids (Chouabia et al., 2021). Similarly, the medicinal compounds ginsenosides in *P. ginseng* exhibit antioxidant activity (Abd-Ellaah et al., 2024).

Evidence from a single study suggests that rosemary extract may help protect the reproductive system against pyrethroid-induced damage, an effect attributed to bioactive antioxidant components that reduce ROS levels and reinforce protective mechanisms (Ali Hasan and Al-Rikaby, 2023).

The anti-inflammatory and antioxidant compounds resveratrol, quercetin, and curcumin have been associated with protective effects against reproductive damage in the studies reviewed (Sharma et al., 2014; 2018).

### 4.4 Synergistic effects and enhanced delivery

In a small number of studies, combining these compounds appeared to enhance protection beyond that of the individual compounds, suggesting a possible synergistic interaction that warrants further confirmation (Sharma et al., 2018; El-Defrawy et al., 2020; Adeniyi et al., 2024). Moreover, a single study reported that chitosan-encapsulated curcumin nanoparticles provided greater protection than conventional curcumin, an effect attributed to improved bioavailability; this finding requires replication across a broader range of natural compounds before firm conclusions can be drawn about nanoparticle-based delivery (Mohamed et al., 2023).

### 4.5 Mechanisms of action of natural products

Natural products exert their protective effects against pyrethroid-induced reproductive toxicity through multiple and interconnected molecular pathways. The main mechanisms identified include enhanced antioxidant defense, improved hormonal axis function, anti-inflammatory responses, and protection of testicular structure. It is important to note that these mechanistic conclusions are derived from a limited subset of the 33 included studies that specifically investigated molecular targets (e.g., Nrf2/HO-1/NQO1, StAR, and NF-κB); most primary studies measured only downstream, non-specific markers such as total antioxidant capacity, MDA, or hormone concentrations without directly probing pathway activation. Consequently, these proposed mechanisms should be interpreted as hypothesis-generating rather than established causal pathways. A further caveat concerns assay validity: several of the most frequently reported bioactive compounds in this review, including curcumin, quercetin, and resveratrol, are well-documented pan-assay interference compounds (PAINS) that can produce non-specific or artefactual readouts in colorimetric and enzyme-based assays through mechanisms such as redox cycling, metal chelation, and non-specific protein reactivity (Bolz et al., 2021).

#### 4.5.1 Antioxidant defense systems

The best-known mechanism involves restoring cellular antioxidant capacity. Pyrethroid exposure significantly reduces the levels of endogenous antioxidant enzymes in testicular tissues (Mohamed et al., 2023). Natural compounds counteract this reduction through multiple pathways. Co-treatment with curcumin and quercetin significantly increased SOD activity and restored CAT activity in cypermethrin-exposed rats, while simultaneously significantly reducing MDA levels (Sharma et al., 2018). Similarly, resveratrol treatment increased glutathione levels and GPx activity in cypermethrin-treated animals (Sharma et al., 2014).

A major molecular target of natural antioxidants is the nuclear factor erythroid-related 2 (Nrf2) signaling pathway (Khan et al., 2024). Under oxidative stress conditions, Nrf2 translocates to the nucleus and binds to antioxidant response elements (AREs), initiating transcription of cytoprotective genes including heme oxygenase-1 (HO-1), NAD(P)H quinone oxidoreductase 1 (NQO1), and GST (Khan et al., 2024). Sage extract can activate Nrf2 signaling, increasing HO-1 expression and NQO1 activity in pyrethroid-exposed testicular tissues (Chouabia et al., 2021).

#### 4.5.2 Hormonal regulation

Natural products restore the function of the hypothalamic-pituitary-gonadal (HPG) axis through several mechanisms. Pyrethroid exposure disrupts steroidogenesis by interfering with the key enzymes 3β-hydroxysteroid dehydrogenase (3β-HSD) and 17β-hydroxysteroid dehydrogenase (17β-HSD) (Sharma et al., 2018). Treatment with *P. ginseng* restored testosterone synthesis by increasing the expression of steroidogenic acute regulatory protein (StAR) and increasing 3β-HSD activity in cypermethrin-exposed rats (Abd-Ellaah et al., 2024). Ginsenosides in *P. ginseng* also increase luteinizing hormone receptor (LHR) expression in Leydig cells and facilitate testosterone biosynthesis (Abd-Ellaah et al., 2024).

*Tribulus terrestris* significantly affected hormonal regulation, increasing testosterone, LH, and FSH levels compared with groups treated with pyrethroid alone (Sharma et al., 2013). The active compounds, particularly protodioscin and protogracilin, appear to stimulate hypothalamic GnRH secretion and enhance pituitary responsiveness to gonadotropin-releasing signals (Sharma et al., 2013).

In this review, studies reported different hormonal responses. These discrepancies may reflect differences in species, pyrethroid type, dose, exposure duration, assay methods, and experimental conditions, as well as methodological heterogeneity.

#### 4.5.3 Anti-inflammatory pathways

A secondary mechanism of pyrethroid-induced reproductive toxicity is chronic inflammation. Cypermethrin exposure increased the levels of proinflammatory cytokines tumor necrosis factor alpha (TNF-α), interleukin-1 beta (IL-1β), and interleukin-6 (IL-6) in the testis (Mohamed et al., 2023). Curcumin treatment significantly attenuated this inflammatory response and reduced TNF-α and IL-1β levels by inhibiting nuclear factor-κB (NF-κB) signaling (Mohamed et al., 2023). The anti-inflammatory effects were mediated by suppression of IκB kinase (IKK) phosphorylation and subsequent prevention of NF-κB nuclear translocation (Mohamed et al., 2023).

#### 4.5.4 Cellular protection and DNA integrity

Natural antioxidants protect against DNA fragmentation and cell apoptosis induced by pyrethroid exposure. Treatment with deltamethrin increased sperm DNA fragmentation and the number of apoptotic cells in the testis (Ben Slima et al., 2013). Treatment with the essential oil of *P. graveolens* reduced DNA fragmentation and the number of apoptotic cells by increasing the expression of the anti-apoptotic protein Bcl-2 and decreasing the expression of the pro-apoptotic proteins Bax and caspase-3 (Ben Slima et al., 2013). Monoterpenes in this oil, especially citronellol and geraniol, exhibited direct free-radical scavenging activity, with IC_50_ values of 15.2 and 18.7 μg/mL, respectively (Ben Slima et al., 2013).

Despite mostly positive findings, several studies report conflicting results that warrant closer investigation. Date palm (*Phoenix dactylifera* L. [Arecaceae]) fruit extract paradoxically reduced testosterone and FSH, despite improving sperm concentration and motility in rats exposed to cypermethrin (Ubah et al., 2021). This suggests a potential dual effect, with antioxidant protection occurring alongside endocrine disruption, possibly due to phytoestrogen content or dose-dependent biphasic responses. This study used a relatively high dose (400 mg/kg) that may have exceeded the therapeutic range, highlighting the critical importance of dose optimization in natural product research (Ubah et al., 2021).

Similarly, *Allium sativum* treatment failed to provide significant protection against deltamethrin-induced reproductive toxicity, and no improvement in sperm parameters or antioxidant enzyme activity was observed compared with the control group that received deltamethrin alone (Desai et al., 2015). This negative result may be attributed to inadequate standardization of bioactive compounds, as this study used crude garlic extract without quantification of key organosulfur compounds such as allicin and diallyl disulfide, which mediate antioxidant effects (Desai et al., 2015). Furthermore, given the 56-day spermatogenic cycle in mice, the 28-day treatment period may not have been long enough to produce observable improvements in reproduction.

The variability in protective efficacy across different natural products and *in vitro* conditions highlights the complexity of pyrethroid–natural product interactions. Factors such as extraction methods, bioactive compound concentrations, treatment duration, dosage regimens, and pyrethroid type significantly influence treatment outcomes. This heterogeneity requires standardized experimental protocols and comprehensive characterization of bioactive compounds to enable reliable intercomparisons and clinical translation.

## 5 Limitations of studies included

Several constraints remain, despite promising evidence that natural compounds protect reproductive health against pyrethroid-induced defects.

### 5.1 Predominance of animal studies

Most investigations to date have used laboratory animals as their primary test subjects. Experimental data from different species create elusive barriers to translating laboratory results to human scientific investigation. We have yet to determine how well the animal-based research findings translate to human reproductive health examination. Notably, no *in vitro* mechanistic or human clinical studies met our eligibility criteria; the evidence base therefore spans only a single tier (*in vivo* animal experiments), and conclusions should be read as applicable to that tier alone until corroborated by mechanistic *in vitro* work and clinical data.

### 5.2 Variability in experimental parameters

Differentiating optimal therapeutic methods is challenging because trials use varying doses, formulation strengths, and treatment durations.

### 5.3 Limited research on specific pyrethroids

Most existing studies evaluate only a limited set of pyrethroid variants, while researchers have not yet examined the influence of the remaining commonly used versions.

### 5.4 Need for standardization in research

The lack of standardized procedures for evaluating reproductive toxicity and protecting natural compounds creates challenges for comparing results across studies. The absence of harmonized protocols for assessing reproductive outcomes and antioxidant markers limits comparability and prevents reliable meta-analyses.

### 5.5 Insufficient understanding of molecular mechanisms

Despite their well-established antioxidant properties, little research explains how natural products function as antioxidants. Research must expand to elucidate how these substances modulate DNA repair processes, cellular signaling, and apoptotic control pathways during pyrethroid exposure.

### 5.6 Assay validity and risk of false-positive findings

Several of the most frequently studied compounds in this review (curcumin, quercetin, resveratrol) are recognized PAINS that can generate non-specific or artefactual signals in colorimetric and enzyme-linked assays (Bolz et al., 2021). Because most primary studies relied on such assays (e.g., spectrophotometric SOD/CAT/GPx activity, MDA/TBARS, ELISA) rather than orthogonal or clinical validation, some reported protective effects might partly reflect assay interference rather than genuine biological activity. This review did not formally grade the certainty of evidence for each outcome domain; future syntheses should adopt a structured level-of-evidence framework to distinguish robust findings from preliminary or speculative ones and to make evidentiary gaps and potential false positives explicit.

Consistent with this concern, a formal risk-of-bias assessment of all 33 included studies using SYRCLE’s Risk of Bias tool (Hooijmans et al., 2014) found that allocation concealment, baseline comparability, random housing, blinding of caregivers/investigators, and random outcome assessment were unclear (i.e., not reported) in 100% of studies, and blinding of the outcome assessor was reported in only one study. In contrast, incomplete outcome data and selective outcome reporting were low risk in the large majority of studies (97%). This profile indicates that the primary evidence base is not necessarily high risk of bias, but that its internal validity is largely unverifiable from the published methods sections, which further supports treating the mechanistic and protective-effect findings summarized in this review as preliminary rather than firmly established.

### 5.7 Review-level limitations

In addition to the limitations of the primary studies, this systematic review has several inherent limitations. First, excluding non-English articles may have introduced language bias, potentially omitting relevant studies published in other languages. Second, despite a comprehensive search, publication bias cannot be excluded, as studies with positive findings are more likely to be published. Finally, the considerable heterogeneity in experimental designs, animal models, dosages, and treatment durations across the included studies precluded a quantitative meta-analysis, limiting the statistical power of our conclusions.

## 6 Future directions

Future research must accomplish several things to overcome these issues.

1. Medical studies should conduct research that involves human participants rather than animals to validate how natural products protect reproductive systems from pyrethroid toxicity.
2. The scientific community should standardize experimental protocols, which include dosage administration procedures, preparation methods, and testing periods, to enhance study comparison reliability.
3. A wider inspection of the most popular pyrethroids in the market should happen to develop complete knowledge about their reproductive stress effects.
4. The specific molecular pathways that natural products employ to reduce oxidative stress and manage apoptotic pathways, as well as other protective effects, need detailed investigation.

## 7 Conclusion

Addressing pyrethroid exposure remains an important public health priority, given the reproductive health risks it poses to exposed individuals in both occupational and environmental settings. The combined effects of oxidative stress, disruption of the hypothalamic-pituitary-gonadal axis, and hormonal imbalance appear to underlie the impairment in sperm quality observed across the reviewed studies.

Natural products rich in bioactive compounds and antioxidants may confer protection against pyrethroid-induced damage in experimental animal models. In a limited number of studies, nanoparticle-based delivery further improved the bioavailability of these compounds, although this evidence remains preliminary and warrants confirmation in additional studies.

The reviewed evidence suggests that natural compounds with antioxidant and hormone-modulating properties may serve as adjunctive strategies against pyrethroid-induced reproductive toxicity, potentially acting through complementary mechanisms; however, dedicated combination studies are needed to confirm this. Future research should pursue translational and, eventually, clinical studies to verify these findings and to develop optimal combination strategies and targeted delivery approaches. Addressing these knowledge gaps will be important for evaluating the potential of natural compounds as protective strategies against environmentally induced reproductive toxicity.

## Data Availability

The raw data supporting the conclusions of this article will be made available by the authors, without undue reservation.

## Glossary

3β-HSD: 3β-hydroxysteroid dehydrogenase
17β-HSD: 17β-hydroxysteroid dehydrogenase
AREs: antioxidant response elements
ARRIVE: animal research reporting *in vivo* experiments
BPA: bisphenol A
CAT: catalase
CYP: cypermethrin
DEL: deltamethrin
DNA: deoxyribonucleic acid
DPACA: diphenylamine colorimetric assay
EDCs: endocrine disrupting chemicals
ELISA: enzyme-linked immunosorbent assay
EPA: Environmental Protection Agency
FEN: fenvalerate
FNP: fenpropathrin
FSH: follicle-stimulating hormone
GnRH: gonadotropin-releasing hormone
GPx: glutathione peroxidase
GR: glutathione reductase
GSH: glutathione
GST: glutathione S-transferase
HO-1: heme oxygenase-1
HPG: hypothalamic-pituitary-gonadal
IHC: immunohistochemical staining
IKK: IκB kinase
IL-1β: Interleukin-1 beta
IL-6: interleukin-6
LH: luteinizing hormone
LHR: luteinizing hormone receptor
LPO: Lipid peroxidation
MDA: malondialdehyde
MeSHs: medical subject headings
NF-κB: nuclear factor-κB
NQO1: NAD(P)H quinone oxidoreductase 1
Nrf2: nuclear factor erythroid-related 2
PCC: protein carbonyl content
PER: permethrin
PICO: population, intervention, comparator, and outcome
PRISMA: preferred reporting items for systematic reviews and meta-analyses
RIA: Radioimmunoassay
ROS: reactive oxygen species
RT-PCR: Real-time polymerase chain reaction
SOD: superoxide dismutase
SPs: synthetic pyrethroids
StAR: steroidogenic acute regulatory protein
SYRCLE: systematic review centre for laboratory animal experimentation
TBARS: thiobarbituric acid-reactive substances
TNF-α: Tumor Necrosis Factor alpha
WHO: World Health Organization

## References

[B1] Abd-Ellaah A. M. Srour S. M. Abdou R. H. Kawther E. (2024). Competence of Panax ginseng on Male fertility in cypermethrin exposed rats. J. Adv. Vet. Res. 13, 2188–2194.

[B2] Adeniyi T. Moronkrji A. Eyovwerhuvu D. (2024). Effects of pyrethroid exposure on testicular histopathology and biochemistry in adult Male wistar rats: evaluation of ascorbic acid and alpha-tocopherol administration. J. Adv. Pharm. Res. 8, 29–37. 10.21608/aprh.2023.234710.1235

[B3] Agarwal A. Baskaran S. Parekh N. Cho C.-L. Henkel R. Vij S. (2021). Male infertility. Lancet 397, 319–333. 10.1016/s0140-6736(20)32667-2 33308486

[B4] Ahamad A. Kumar J. (2023). Pyrethroid pesticides: an overview on classification, toxicological assessment and monitoring. J. Hazard. Mater. Adv. 10, 100284. 10.1016/j.hazadv.2023.100284

[B5] Ali Hasan S. Al-Rikaby A. A. (2023). Evaluating the influence of rosemary leaves extract on hormonal and histopathological alterations in Male rabbits exposed to cypermethrin. Arch. Razi Inst. 78, 797–805. 10.22092/ARI.2022.359859.2487 38028826 PMC10657936

[B6] Atef M. M. El-Deeb O. S. Sadek M. T. Abo El Gheit R. E. Emam M. N. Hafez Y. M. (2020). Targeting ERK/COX-2 signaling pathway in permethrin-induced testicular toxicity: a possible modulating effect of matrine. Mol. Biol. Rep. 47, 247–259. 10.1007/s11033-019-05125-7 31621018

[B7] Bagherpour H. Karimpour Malekshah A. Talebpour Amiri F. Azadbakht M. (2019). Protective effect of green tea extract on the deltamethrin-induced toxicity in mice testis: an experimental study. Int. J. Reprod. Biomed. 17, 337–348. 10.18502/ijrm.v17i5.4601 31435613 PMC6653493

[B8] Barr D. B. Olsson A. O. Wong L.-Y. Udunka S. Baker S. E. Whitehead R. D. (2010). Urinary concentrations of metabolites of pyrethroid insecticides in the general U.S. population: National health and nutrition examination survey 1999-2002. Environ. Health Perspect. 118, 742–748. 10.1289/ehp.0901275 20129874 PMC2898848

[B9] Ben Halima N. Ben Slima A. Moalla I. Fetoui H. Pichon C. Gdoura R. (2014). Protective effects of oat oil on deltamethrin-induced reprotoxicity in Male mice. Food Funct. 5, 2070–2077. 10.1039/C4FO00190G 24992227

[B10] Ben Slima A. Ali M. B. Barkallah M. Traore A. I. Boudawara T. Allouche N. (2013). Antioxidant properties of Pelargonium graveolens l’Her essential oil on the reproductive damage induced by deltamethrin in mice as compared to alpha-tocopherol. Lipids Health Dis. 12, 30. 10.1186/1476-511X-12-30 23496944 PMC3641007

[B11] Bhattacharya I. Sharma S. S. Majumdar S. S. (2024). Etiology of Male infertility: an update. Reprod. Sci. 31, 942–965. 10.1007/s43032-023-01401-x 38036863

[B12] Bolz S. N. Adasme M. F. Schroeder M. (2021). Toward an understanding of pan-assay interference compounds and promiscuity: a structural perspective on binding modes. J. Chem. Inf. Model. 61 (5), 2248–2262. 10.1021/acs.jcim.0c01227 33899463

[B13] Chouabia A. Djemli S. Abdennour C. Mallem L. Kahalerras L. Arkoub F. Z. (2021). Protective effect of Salvia officinalis against cypermethrin-induced reprotoxicity in male wistar rats. Pharmacogn. J. 13, 1413–1421. 10.5530/pj.2021.13.179

[B14] Darwish S. F. Moustafa Y. M. Abdel Mageed S. S. Hassan G. S. Mangoura S. A. Aly S. H. (2025). Insecticides and testicular health: mechanisms of injury and protective natural products. Naunyn-Schmiedeberg's Archives Pharmacol. 398, 11229–11259. 10.1007/s00210-025-04016-y 40137965 PMC12449350

[B15] Desai K. R. Moid N. Patel P. B. Highland H. N. (2015). Protective efficacy of Allium sativum on deltamethrin induced toxicity in reproductive tissues of male mice. Int. J. Pharm. Sci. Res. 6, 1711.

[B16] El-Defrawy S. H. S. Sobhy H. M. El-Kholy T. A. El-Hashash S. A. Sultan H. A. (2020). Protective effect of moringa and sage on fertility impairment induced by synthetic pyrethroids in Male rats. Egypt. J. Chem. Environ. heal. 6, 13–27. 10.21608/ejceh.2020.238155

[B17] El-Demerdash F. M. Yousef M. I. Kedwany F. S. Baghdadi H. H. (2004). Role of α-Tocopherol and β-Carotene in ameliorating the fenvalerate-induced changes in oxidative stress, hemato-biochemical parameters, and semen quality of Male rats. J. Environ. Sci. Heal. Part B 39, 443–459. 10.1081/PFC-120035929 15186033

[B18] El‐Demerdash F. Yousef M. AL‐Salhen K. (2003). Protective effects of isoflavone on some biochemical parameters affected by cypermethrin in male rabbits. J. Environ. Sci. Heal. Part B 38, 365–378. 10.1081/PFC-120019902 12716053

[B19] Fenner K. Canonica S. Wackett L. P. Elsner M. (2013). Evaluating pesticide degradation in the environment: blind spots and emerging opportunities. Sci. 341, 752–758. 10.1126/science.1236281 23950532

[B20] Herman A. Constantin M. Colombe D. D. R. Gestil N. T. D. Ferry M. J. M'boko M. (2024). Evaluation of the pharmacological potential of Strychnos camptoneura trunk bark hydroethanol extract on reproductive functions in male Guinea pigs (*Cavia porcellus*) exposed to cypermethrin. cabidigitallibrary.Org. 15, 884–898. 10.4236/ajps.2024.1510057

[B21] Hooijmans C. R. de Vries R. Leenaars M. Curfs J. Ritskes‐Hoitinga M. (2011). Improving planning, design, reporting and scientific quality of animal experiments by using The Gold standard publication checklist, in addition to the ARRIVE guidelines. Br. J. Pharmacol. 162, 1259–1260. 10.1111/j.1476-5381.2010.01128.x 21091655 PMC3058159

[B22] Hooijmans C. R. Rovers M. M. De Vries R. B. Leenaars M. Ritskes-Hoitinga M. Langendam M. W. (2014). SYRCLE’s risk of bias tool for animal studies. BMC Medical Research Methodology 14 (1), 43. 10.1186/1471-2288-14-43 24667063 PMC4230647

[B23] Jebur A. B. El-Sayed R. A. Abdel-Daim M. M. El-Demerdash F. M. (2023a). Punica granatum (pomegranate) peel extract pre-treatment alleviates fenpropathrin-induced testicular injury *via* suppression of oxidative stress and inflammation in adult Male rats. Toxics 11, 504. 10.3390/toxics11060504 37368604 PMC10301163

[B24] Jebur A. El-Sayed R. El-Demerdash F. (2023b). Lycopene modulates testicular injury oxidative stress and caspases upregulation induced by fenvalerate in Male rats. Bionatura 8, 1–15. 10.21931/RB/2023.08.04.76

[B25] Kale M. Rathore N. John S. Bhatnagar D. (1999). Lipid peroxidative damage on pyrethroid exposure and alterations in antioxidant status in rat erythrocytes: a possible involvement of reactive oxygen species. Toxicol. Lett. 105, 197–205. 10.1016/S0378-4274(98)00399-3 10355540

[B26] Khaki A. Khaki A. A. Rajabzadeh A. (2017). The effects of permethrin and antioxidant properties of Allium cepa (onion) on testicles parameters of male rats. Toxin Rev. 36, 1–6. 10.1080/15569543.2016.1235582

[B27] Khan M. Z. Khan A. Huang B. Wei R. Kou X. Wang X. (2024). Bioactive compounds protect mammalian reproductive cells from xenobiotics and heat stress-induced oxidative distress *via* Nrf2 signaling activation: a narrative review. Antioxidants 13, 597. 10.3390/antiox13050597 38790702 PMC11118937

[B28] Khormi M. A. Alfattah M. A. Abo-Zaid M. A. Abdalla S. E. B. El-Shabasy A. , (2025). Alleviation potential activity of cypermethrin by Moringa oleifera lam. oil on testes and livers of male rats with response to affinity of specific physiological protein. Asian J. Agri &Bio 10.35495/ajab.2024.160

[B29] Luo J. Leng W. D. (2013). Theory and practice of systematic review/meta-analysis. Chin. J. Evidence-Based Cardiovasc. Med. 5, 115p. 10.1016/j.fct.2023.113977

[B30] Mohamed A. A.-R. Behairy A. Abd El-Hakim Y. M. Metwally M. M. M. Khamis T. Abuzahrah S. S. (2023). Comparable bio-evaluation of curcumin and chitosan-encapsulated curcumin nanoparticles against the reprotoxic potential of fenpropathrin pyrethroid in rats: genomic and morphometric prospectives. Food Chem. Toxicol. 179, 113977. 10.1016/j.fct.2023.13977 37532170

[B31] Moher D. Liberati A. Tetzlaff J. Altman D. G. (2009). Preferred reporting items for systematic reviews and meta-analyses: the PRISMA statement. BMJ 339, b2535. 10.1136/bmj.b2535 19622551 PMC2714657

[B32] Narcisse V. B. Augustave K. Zaki N. N. B. Ferdinand N. Edouard N. A. Martine T. M. A. (2017). Protective effects of the aqueous extract of Bersama engleriana leaves against cypermethrin-induced oxidative stress and reproductive toxicity in Male Guinea pig (*Cavia porcellus*). Int. J. Innov. Appl. Stud. 22, 78–85.

[B33] Nashed M. S. Hassanen E. I. Issa M. Y. Tohamy A. F. Prince A. M. Hussien A. M. (2024). The mollifying effect of Sambucus nigra extract on StAR gene expression, oxidative stress, and apoptosis induced by fenpropathrin in male rats. Food Chem. Toxicol. 189, 114744. 10.1016/j.fct.2024.114744 38782235

[B34] Oda S. S. El-Maddawy Z. K. (2012). Protective effect of vitamin E and selenium combination on deltamethrin-induced reproductive toxicity in male rats. Exp. Toxicol. Pathol. 64, 813–819. 10.1016/j.etp.2011.03.001 21478004

[B35] Osama E. Galal A. A. A. Abdalla H. El-Sheikh S. M. A. (2019). Chlorella vulgaris ameliorates testicular toxicity induced by deltamethrin in male rats *via* modulating oxidative stress. Andrologia 51, e13214. 10.1111/and.13214 30488469

[B36] Page M. J. McKenzie J. E. Bossuyt P. M. Boutron I. Hoffmann T. C. Mulrow C. D. (2021). The PRISMA 2020 statement: an updated guideline for reporting systematic reviews. Bmj, 372. n71. 10.1136/bmj.n71 33782057 PMC8005924

[B37] Radi A. M. Abdel-Azeem N. M. Mostafa I. Helmy N. A. Ahmed W. (2021). The protective role of wheat germ oil against adverse effect of deltamethrin on reproductive aspects of male albino rats. J. Appl. Vet. Sci. 6, 70–75.

[B38] Rajabzadeh A. Khaki A. A. Khaki A. (2018). Antioxidant properties of Allium cepa (onion) against permethrin-induced toxicity on LHCGR and SF1 genes in Male rats. Crescent J. Med. Biol. Sci. 5, 327–331.

[B39] Sakr S. A. Al-Amoudi W. M. (2012). Effect of ginger extract on deltamethrin induced histomorphological and immunohistochemical changes in testes of albino rats. Life Sci. J. 9, 771–778.

[B40] Sengupta P. Banerjee R. (2014). Environmental toxins: alarming impacts of pesticides on male fertility. Hum. Exp. Toxicol. 33, 1017–1039. 10.1177/0960327113515504 24347299

[B41] Sharlip I. D. Jarow J. P. Belker A. M. Lipshultz L. I. Sigman M. Thomas A. J. (2002). Best practice policies for male infertility. Fertil. Steril. 77, 873–882. 10.1016/S0015-0282(02)03105-9 12009338

[B42] Sharma P. Huq A. U. Singh R. (2013). Cypermethrin induced reproductive toxicity in male wistar rats: protective role of *Tribulus terristris* . J. Environ. Biol. 34, 857–862. 24558798

[B43] Sharma P. Huq A. Singh R. (2014). Cypermethrin-induced reproductive toxicity in the rat is prevented by resveratrol. J. Hum. Reprod. Sci. 7, 99–106. 10.4103/0974-1208.138867 25191022 PMC4150150

[B44] Sharma P. Khan I. Singh R. (2016). Efficacy of Curculigo orchioides in deltamethrin induced reproductive system impairment in male wistar rats. Asian J. Pharm. 10, S100–S109.

[B45] Sharma P. Aslam Khan I. Singh R. (2018). Curcumin and quercetin ameliorated cypermethrin and deltamethrin-induced reproductive system impairment in Male wistar rats by upregulating the activity of pituitary-gonadal hormones and steroidogenic enzymes. Int. J. Fertil. Steril. 12, 72–80. 10.22074/ijfs.2018.5160 29334211 PMC5767937

[B46] Soliman M. M. Nashed M. S. Hassanen E. I. Issa M. Y. Prince A. M. Hussien A. M. (2025). Ameliorative effects of date palm kernel extract against fenpropathrin induced male reproductive toxicity. Biol. Res. 58, 27. 10.1186/s40659-025-00605-6 40329351 PMC12057013

[B47] Tanushreea A. K. Kumara A. (2024). Study on mitigating role of fenugreek aqueous extract against gonadotoxic induced by permethrin on male swiss albino mice (mus musculus). Biospectra 19, 57–60. 10.5281/zenodo.14263339

[B48] Ubah S. A. Agbonu O. A. Columbus P. K. Abah K. O. Chibuogwu I. C. Abalaka S. E. (2021). Effects of date fruit (phoenix dactylifera) on sperm cell morphology and reproductive hormonal profiles in cypermethrin-induced male infertility in wister rats. Sci. Afr. 11, e00713. 10.1016/j.sciaf.2021.e00713

[B49] Vemo B. N. Kenfack A. Ngoula F. Nantia E. A. Kodjio N. Guiekep A. J. N. (2017). Effects of ethanol extract of Bersama engleriana leaves on oxidative stress and reproductive parameters in male Guinea pig (Cavia porcellus) exposed to cypermethrin. Int. J. Biol. Chem. Sci. 11, 2243–2253. 10.4314/ijbcs.v11i5.23

[B50] Wang H. Yang F. Ye J. Dai X. Liao H. Xing C. (2024). Ginkgo biloba extract alleviates deltamethrin-induced testicular injury by upregulating SKP2 and inhibiting Beclin1-independent autophagy. Phytomedicine 135, 156245. 10.1016/j.phymed.2024.156245 39550920

[B51] Yao K.-W. Wang J.-D. (2008). Progress in studies of the male reproductive toxicity of pyrethroid insecticides. Zhonghua Nan Ke Xue= Natl. J. Androl. 14, 268–271. 18488346

[B52] Ye X. Liu J. (2019). Effects of pyrethroid insecticides on hypothalamic-pituitary-gonadal axis: a reproductive health perspective. Environ. Pollut. 245, 590–599. 10.1016/j.envpol.2018.11.031 30476888

[B53] Yousef M. I. El‐Demerdash F. M. Al‐Salhen K. S. (2003). Protective role of isoflavones against the toxic effect of cypermethrin on semen quality and testosterone levels of rabbits. J. Environ. Sci. Heal. Part B 38, 463–478. 10.1081/PFC-120021666 12856928

[B54] Zhang J. Zhang J. Liu R. Gan J. Liu J. Liu W. (2016). Endocrine-disrupting effects of pesticides through interference with human glucocorticoid receptor. Environ. Sci. Technol. 50, 435–443. 10.1021/acs.est.5b03731 26647222

