# Supplementary material for: Natural products mitigate pyrethroid-induced male reproductive toxicity: a systematic review
Source: Front Pharmacol. 2026 Oct 26;17:1933092. doi: 10.3389/fphar.2026.1933092 (PMC13745655; doi:10.3389/fphar.2026.1933092)
Supplement: Supplementary file 1 [file Table2.docx]

| **Table S2.** Search strategies including the key terms and the queries for each database | |
| --- | --- |
| **Database**  July 2025 | **key terms and the queries** |
| PubMed | ("Infertility"[MeSH Terms] OR "Infertility"[Title/Abstract] OR "Reproductive Sterility"[Title/Abstract] OR "Sterility"[Title/Abstract] OR "Subfertility"[Title/Abstract] OR ("Infertility"[MeSH Terms] OR "Infertility"[Title/Abstract] OR "Reproductive Sterility"[Title/Abstract] OR "Sterility"[Title/Abstract] OR "Subfertility"[Title/Abstract] OR "Subfertile"[Title/Abstract]) OR ("Azoospermia"[MeSH Terms] OR "Azoospermia"[Title/Abstract]) OR ("Oligospermia"[MeSH Terms] OR "Oligospermia"[Title/Abstract] OR "Oligoasthenoteratozoospermia"[Title/Abstract] OR "Oligoasthenoteratozoospermias"[Title/Abstract] OR "Low Sperm Count"[Title/Abstract] OR "Oligozoospermia"[Title/Abstract] OR "Low Sperm Counts"[Title/Abstract] OR "Hypospermatogenesis"[Title/Abstract] OR "Cryptozoospermia"[Title/Abstract] OR "Cryptospermia"[Title/Abstract] OR "Cryptospermias"[Title/Abstract]) OR ("Genitalia"[MeSH Terms] OR "Genitalia"[Title/Abstract] OR "genital disease"[Title/Abstract]) OR "Sperm count"[Title/Abstract] OR "Low Sperm Count"[Title/Abstract] OR "sperm concentration"[Title/Abstract] OR "sperm morphology"[Title/Abstract] OR "sperm motility"[Title/Abstract] OR "sperm fertilization capacity"[Title/Abstract] OR "follicle stimulating hormone"[Title/Abstract] OR "luteinizing hormone"[Title/Abstract] OR "testis size"[Title/Abstract] OR "sexual drive"[Title/Abstract] OR "Sperm toxicity"[Title/Abstract] OR "testis toxicity"[Title/Abstract] OR "testis histology"[Title/Abstract]) AND ("Herbal Medicine"[MeSH Terms] OR "Herbal Medicine"[Title/Abstract] OR "Herbalism"[Title/Abstract] OR ("Plant Extracts"[MeSH Terms] OR "Plant Extracts"[Title/Abstract] OR "Plant Extract"[Title/Abstract]) OR ("Homeopathy"[MeSH Terms] OR "Homeopathy"[Title/Abstract]) OR ("plants, medicinal"[MeSH Terms] OR "Medicinal Plants"[Title/Abstract] OR "Medicinal Plant"[Title/Abstract] OR "Medicinal Herbs"[Title/Abstract] OR "Herb Medicinal"[Title/Abstract] OR "Pharmaceutical Plants"[Title/Abstract] OR "Pharmaceutical Plant"[Title/Abstract] OR "Healing Plant"[Title/Abstract] OR "Healing Plants"[Title/Abstract]) OR ("medicine, traditional"[MeSH Terms] OR "Traditional Medicine"[Title/Abstract] OR "Folk Medicine"[Title/Abstract] OR "Indigenous Medicine"[Title/Abstract] OR "Ethnomedicine"[Title/Abstract] OR "Folk Remedies"[Title/Abstract]) OR ("Biological Products"[MeSH Terms] OR "Biological Products"[Title/Abstract] OR "Natural Products"[Title/Abstract] OR "Natural Product"[Title/Abstract]) OR ("Phytotherapy"[MeSH Terms] OR "Phytotherapy"[Title/Abstract] OR "Herb Therapy"[Title/Abstract] OR "Herbal Therapy"[Title/Abstract]) OR ("medicine, persian"[MeSH Terms] OR "Persian Medicine"[Title/Abstract] OR "Persian Traditional Medicine"[Title/Abstract] OR "Iranian Traditional Medicine"[Title/Abstract] OR "Ancient Iranian Medicine"[Title/Abstract]) OR ("medicine, chinese traditional"[MeSH Terms] OR "Chinese Traditional Medicine"[Title/Abstract])) AND ("Pyrethrins"[MeSH Terms] OR "Pyrethrins"[Title/Abstract] OR "Pyrethrin"[Title/Abstract] OR "Pyrethroids"[Title/Abstract] OR "Pyrethroid"[Title/Abstract] OR ("Permethrin"[MeSH Terms] OR "Permethrin"[Title/Abstract] OR "Permethrin"[Title/Abstract]) OR "bifenthrin"[Title/Abstract] OR "beta-cyfluthrin"[Title/Abstract] OR "cyfluthrin"[Title/Abstract] OR "cyphenothrin"[Title/Abstract] OR "deltamethrin"[Title/Abstract] OR "esfenvalerate"[Title/Abstract] OR "etofenprox"[Title/Abstract] OR "fenpropathrin"[Title/Abstract] OR "taufluvalinate"[Title/Abstract] OR "lambda cyhalothrin"[Title/Abstract] OR "gamma cyhalothrin"[Title/Abstract] OR "imiprothrin"[Title/Abstract] OR "prallethrin"[Title/Abstract] OR "prallethrin"[Title/Abstract] OR "resmethrin"[Title/Abstract] OR "sumithrin"[Title/Abstract] OR "d-phenothrin"[Title/Abstract] OR "tefluthrin"[Title/Abstract] OR "tralomethrin"[Title/Abstract] OR "zeta-cypermethrin"[Title/Abstract] OR "cypermethrin"[Title/Abstract]) |
| Web of Science (ISI) | Query #1  "Pyrethrins" (Topic) or "Pyrethrin" (Topic) or "Pyrethroids" (Topic) or "Pyrethroid" (Topic) or "Permethrin" (Topic) or "allethrin stereoisomers" (Topic) or "bifenthrin" (Topic) or "beta-cyfluthrin" (Topic) or "cyfluthrin" (Topic) or "cyphenothrin" (Topic) or "deltamethrin" (Topic) or "esfenvalerate" (Topic) or "etofenprox" (Topic) or "fenpropathrin" (Topic) or "taufluvalinate" (Topic) or "lambda cyhalothrin" (Topic) or "gamma cyhalothrin" (Topic) or "imiprothrin" (Topic) or "1RS cispermethrin" (Topic) or "prallethrin" (Topic) or "resmethrin" (Topic) or "sumithrin" (Topic) or "d-phenothrin" (Topic) or "tefluthrin" (Topic) or "tralomethrin" (Topic) or "zeta-cypermethrin" (Topic) or "cypermethrin" (Topic)  Query #2  "Herbal Medicine" (Topic) or "Herbalism" (Topic) or "Plant Extracts" (Topic) or "Plant Extract" (Topic) or "Homeopathy" (Topic) or "Plants, Medicinal" (Topic) or "Medicinal Plants" (Topic) or "Medicinal Plant" (Topic) or "Medicinal Herbs" (Topic) or "Herb Medicinal" (Topic) or "Pharmaceutical Plants" (Topic) or "Pharmaceutical Plant" (Topic) or "Healing Plant" (Topic) or "Healing Plants" (Topic) or "Medicine, Traditional" (Topic) or "Traditional Medicine" (Topic) or "Folk Medicine" (Topic) or "Indigenous Medicine" (Topic) or "Ethnomedicine" (Topic) or "Folk Remedies" (Topic) or "Biological Products" (Topic) or "Natural Products" (Topic) or "Natural Product" (Topic) or "Phytotherapy" (Topic) or "Herb Therapy" (Topic) or "Herbal Therapy" (Topic) or "Medicine, Persian" (Topic) or "Persian Medicine" (Topic) or "Persian Traditional Medicine" (Topic) or "Iranian Traditional Medicine" (Topic) or "Ancient Iranian Medicine" (Topic) or "Medicine, Chinese Traditional" (Topic) or "Chinese Traditional Medicine" (Topic)  Query #3  "Infertility" (Topic) or "Reproductive Sterility" (Topic) or "Sterility" (Topic) or "Subfertility" (Topic) or "Infertility" (Topic) or "Subfertile" (Topic) or "Azoospermia" (Topic) or "Oligospermia" (Topic) or "Oligoasthenoteratozoospermia" (Topic) or "Oligoasthenoteratozoospermias" (Topic) or "Low Sperm Count" (Topic) or "Oligozoospermia" (Topic) or "Low Sperm Counts" (Topic) or "Hypospermatogenesis" (Topic) or "Cryptozoospermia" (Topic) or "Cryptospermia" (Topic) or "Cryptospermias" (Topic) or "Genitalia" (Topic) or "genital disease" (Topic) or "Sperm count" (Topic) or "sperm concentration" (Topic) or "sperm morphology" (Topic) or "sperm motility" (Topic) or "sperm fertilization capacity" (Topic) or "sperm capacitation reaction" (Topic) or "follicle stimulating hormone" (Topic) or "luteinizing hormone" (Topic) or "testis size" (Topic) or "testis weigh" (Topic) or "sexual drive" (Topic) or "Sperm toxicity" (Topic) or "testis toxicity" (Topic) or "testis histology" (Topic) |
| Scopus | ( ( TITLE-ABS-KEY ( "Pyrethrins" ) OR TITLE-ABS-KEY ( "Pyrethrin" ) OR TITLE-ABS-KEY ( "Pyrethroids" ) OR TITLE-ABS-KEY ( "Pyrethroid" ) OR TITLE-ABS-KEY ( "Permethrin" ) OR TITLE-ABS-KEY ( "allethrin stereoisomers" ) OR TITLE-ABS-KEY ( "bifenthrin" ) OR TITLE-ABS-KEY ( "beta-cyfluthrin" ) OR TITLE-ABS-KEY ( "cyfluthrin" ) OR TITLE-ABS-KEY ( "cyphenothrin" ) OR TITLE-ABS-KEY ( "deltamethrin" ) OR TITLE-ABS-KEY ( "esfenvalerate" ) OR TITLE-ABS-KEY ( "etofenprox" ) OR TITLE-ABS-KEY ( "fenpropathrin" ) OR TITLE-ABS-KEY ( "taufluvalinate" ) OR TITLE-ABS-KEY ( "lambda cyhalothrin" ) OR TITLE-ABS-KEY ( "gamma cyhalothrin" ) OR TITLE-ABS-KEY ( "imiprothrin" ) OR TITLE-ABS-KEY ( "1RS cispermethrin" ) OR TITLE-ABS-KEY ( "prallethrin" ) OR TITLE-ABS-KEY ( "resmethrin" ) OR TITLE-ABS-KEY ( "sumithrin" ) OR TITLE-ABS-KEY ( "d-phenothrin" ) OR TITLE-ABS-KEY ( "tefluthrin" ) OR TITLE-ABS-KEY ( "tralomethrin" ) OR TITLE-ABS-KEY ( "zeta-cypermethrin" ) OR TITLE-ABS-KEY ( "cypermethrin" ) ) ) AND ( ( TITLE-ABS-KEY ( "Herbal Medicine" ) OR TITLE-ABS-KEY ( "Herbalism" ) OR TITLE-ABS-KEY ( "Plant Extracts" ) OR TITLE-ABS-KEY ( "Plant Extract" ) OR TITLE-ABS-KEY ( "Homeopathy" ) OR TITLE-ABS-KEY ( "Plants, Medicinal" ) OR TITLE-ABS-KEY ( "Medicinal Plants" ) OR TITLE-ABS-KEY ( "Medicinal Plant" ) OR TITLE-ABS-KEY ( "Medicinal Herbs" ) OR TITLE-ABS-KEY ( "Herb Medicinal" ) OR TITLE-ABS-KEY ( "Pharmaceutical Plants" ) OR TITLE-ABS-KEY ( "Pharmaceutical Plant" ) OR TITLE-ABS-KEY ( "Healing Plant" ) OR TITLE-ABS-KEY ( "Healing Plants" ) OR TITLE-ABS-KEY ( "Medicine, Traditional" ) OR TITLE-ABS-KEY ( "Traditional Medicine" ) OR TITLE-ABS-KEY ( "Folk Medicine" ) OR TITLE-ABS-KEY ( "Indigenous Medicine" ) OR TITLE-ABS-KEY ( "Ethnomedicine" ) OR TITLE-ABS-KEY ( "Folk Remedies" ) OR TITLE-ABS-KEY ( "Biological Products" ) OR TITLE-ABS-KEY ( "Natural Products" ) OR TITLE-ABS-KEY ( "Natural Product" ) OR TITLE-ABS-KEY ( "Phytotherapy" ) OR TITLE-ABS-KEY ( "Herb Therapy" ) OR TITLE-ABS-KEY ( "Herbal Therapy" ) OR TITLE-ABS-KEY ( "Medicine, Persian" ) OR TITLE-ABS-KEY ( "Persian Medicine" ) OR TITLE-ABS-KEY ( "Persian Traditional Medicine" ) OR TITLE-ABS-KEY ( "Iranian Traditional Medicine" ) OR TITLE-ABS-KEY ( "Ancient Iranian Medicine" ) OR TITLE-ABS-KEY ( "Medicine, Chinese Traditional" ) OR TITLE-ABS-KEY ( "Chinese Traditional Medicine" ) ) ) AND ( ( TITLE-ABS-KEY ( "Infertility" ) OR TITLE-ABS-KEY ( "Reproductive Sterility" ) OR TITLE-ABS-KEY ( "Sterility" ) OR TITLE-ABS-KEY ( "Subfertility" ) OR TITLE-ABS-KEY ( "Subfertile" ) OR TITLE-ABS-KEY ( "Azoospermia" ) OR TITLE-ABS-KEY ( "Oligospermia" ) OR TITLE-ABS-KEY ( "Oligoasthenoteratozoospermia" ) OR TITLE-ABS-KEY ( "Oligoasthenoteratozoospermias" ) OR TITLE-ABS-KEY ( "Low Sperm Count" ) OR TITLE-ABS-KEY ( "Oligozoospermia" ) OR TITLE-ABS-KEY ( "Low Sperm Counts" ) OR TITLE-ABS-KEY ( "Hypospermatogenesis" ) OR TITLE-ABS-KEY ( "Cryptozoospermia" ) OR TITLE-ABS-KEY ( "Cryptospermia" ) OR TITLE-ABS-KEY ( "Cryptospermias" ) OR TITLE-ABS-KEY ( "Genitalia" ) OR TITLE-ABS-KEY ( "genital disease" ) OR TITLE-ABS-KEY ( "Sperm count" ) OR TITLE-ABS-KEY ( "sperm concentration" ) OR TITLE-ABS-KEY ( "sperm morphology" ) OR TITLE-ABS-KEY ( "sperm motility" ) OR TITLE-ABS-KEY ( "sperm fertilization capacity" ) OR TITLE-ABS-KEY ( "sperm capacitation reaction" ) OR TITLE-ABS-KEY ( "follicle stimulating hormone" ) OR TITLE-ABS-KEY ( "luteinizing hormone" ) OR TITLE-ABS-KEY ( "testis size" ) OR TITLE-ABS-KEY ( "testis weight" ) OR TITLE-ABS-KEY ( "sexual drive" ) OR TITLE-ABS-KEY ( "Sperm toxicity" ) OR TITLE-ABS-KEY ( "testis toxicity" ) OR TITLE-ABS-KEY ( "testis histology" ) ) ) |
| Embase: | Query #1:  (("Pyrethrins":ti,ab,kw OR "Pyrethrin":ti,ab,kw OR "Pyrethroids":ti,ab,kw OR "Pyrethroid":ti,ab,kw OR "Permethrin":ti,ab,kw OR "allethrin stereoisomers":ti,ab,kw OR "bifenthrin":ti,ab,kw OR "beta-cyfluthrin":ti,ab,kw OR "cyfluthrin":ti,ab,kw OR "cyphenothrin":ti,ab,kw OR "deltamethrin":ti,ab,kw OR "esfenvalerate":ti,ab,kw OR "etofenprox":ti,ab,kw OR "fenpropathrin":ti,ab,kw OR "taufluvalinate":ti,ab,kw OR "lambda cyhalothrin":ti,ab,kw OR "gamma cyhalothrin":ti,ab,kw OR "imiprothrin":ti,ab,kw OR "1RS cispermethrin":ti,ab,kw OR "prallethrin":ti,ab,kw OR "resmethrin":ti,ab,kw OR "sumithrin":ti,ab,kw OR "d-phenothrin":ti,ab,kw OR "tefluthrin":ti,ab,kw OR "tralomethrin":ti,ab,kw OR "zeta-cypermethrin":ti,ab,kw OR "cypermethrin":ti,ab,kw))  Query #2:  (("Herbal Medicine":ti,ab,kw OR "Herbalism":ti,ab,kw OR "Plant Extracts":ti,ab,kw OR "Plant Extract":ti,ab,kw OR "Homeopathy":ti,ab,kw OR "Plants, Medicinal":ti,ab,kw OR "Medicinal Plants":ti,ab,kw OR "Medicinal Plant":ti,ab,kw OR "Medicinal Herbs":ti,ab,kw OR "Herb Medicinal":ti,ab,kw OR "Pharmaceutical Plants":ti,ab,kw OR "Pharmaceutical Plant":ti,ab,kw OR "Healing Plant":ti,ab,kw OR "Healing Plants":ti,ab,kw OR "Medicine, Traditional":ti,ab,kw OR "Traditional Medicine":ti,ab,kw OR "Folk Medicine":ti,ab,kw OR "Indigenous Medicine":ti,ab,kw OR "Ethnomedicine":ti,ab,kw OR "Folk Remedies":ti,ab,kw OR "Biological Products":ti,ab,kw OR "Natural Products":ti,ab,kw OR "Natural Product":ti,ab,kw OR "Phytotherapy":ti,ab,kw OR "Herb Therapy":ti,ab,kw OR "Herbal Therapy":ti,ab,kw OR "Medicine, Persian":ti,ab,kw OR "Persian Medicine":ti,ab,kw OR "Persian Traditional Medicine":ti,ab,kw OR "Iranian Traditional Medicine":ti,ab,kw OR "Ancient Iranian Medicine":ti,ab,kw OR "Medicine, Chinese Traditional":ti,ab,kw OR "Chinese Traditional Medicine":ti,ab,kw))  Query #3:  (("Infertility":ti,ab,kw OR "Reproductive Sterility":ti,ab,kw OR "Sterility":ti,ab,kw OR "Subfertility":ti,ab,kw OR "Subfertile":ti,ab,kw OR "Azoospermia":ti,ab,kw OR "Oligospermia":ti,ab,kw OR "Oligoasthenoteratozoospermia":ti,ab,kw OR "Oligoasthenoteratozoospermias":ti,ab,kw OR "Low Sperm Count":ti,ab,kw OR "Oligozoospermia":ti,ab,kw OR "Low Sperm Counts":ti,ab,kw OR "Hypospermatogenesis":ti,ab,kw OR "Cryptozoospermia":ti,ab,kw OR "Cryptospermia":ti,ab,kw OR "Cryptospermias":ti,ab,kw OR "Genitalia":ti,ab,kw OR "genital disease":ti,ab,kw OR "Sperm count":ti,ab,kw OR "sperm concentration":ti,ab,kw OR "sperm morphology":ti,ab,kw OR "sperm motility":ti,ab,kw OR "sperm fertilization capacity":ti,ab,kw OR "sperm capacitation reaction":ti,ab,kw OR "follicle stimulating hormone":ti,ab,kw OR "luteinizing hormone":ti,ab,kw OR "testis size":ti,ab,kw OR "testis weight":ti,ab,kw OR "sexual drive":ti,ab,kw OR "Sperm toxicity":ti,ab,kw OR "testis toxicity":ti,ab,kw OR "testis histology":ti,ab,kw)) |
