# Supplementary material for: Natural products mitigate pyrethroid-induced male reproductive toxicity: a systematic review
Source: Front Pharmacol. 2026 Oct 26;17:1933092. doi: 10.3389/fphar.2026.1933092 (PMC13745655; doi:10.3389/fphar.2026.1933092)
Supplement: Supplementary file 2 [file Table4.docx]

**Table S4.** Quality assessment of animal studies.

|  | Author, Year | Generalizability/ translation | Adverse events | Numbers analysed | Statistical method | Allocating animals to experimental groups | Housing and husbandry | Experimental procedures | Ethical statement | Background | Title | Total Score |
| --- | --- | --- | --- | --- | --- | --- | --- | --- | --- | --- | --- | --- |
| 1 | Abd-Ellaah A. M., et al. 2023. | * | * |  | * | * |  | * |  | * | * | 7 |
| 2 | Adeniyi T., et al. 2024. |  |  | * | * | * | * | * | * | * | * | 9 |
| 3 | Ali Hasan S., and Al-Rikaby A. 2023. | * |  | * | * | * | * | * |  | * | * | 8 |
| 4 | Atef M. M., et al. 2020. | * |  | * | * | * | * | * |  | * | * | 8 |
| 5 | Bagherpour H., et al. 2019. | * |  | * | * | * | * | * | * | * | * | 9 |
| 6 | Ben Halima N., et al. 2014. | * |  | * | * | * | * | * | * | * | * | 9 |
| 7 | Ben Slima A., et al, 2013. | * |  | * | * | * | * | * | * | * | * | 9 |
| 8 | Chouabia A., et al. 2021. | * |  | * | * | * | * | * |  | * | * | 8 |
| 9 | Desai,K., et al. 2015. | * |  |  | * | * |  | * |  | * | * | 6 |
| 10 | El-Defrawy S. H. S., et al. 2020. | * |  | * | * | * | * | * |  | * | * | 8 |
| 11 | El-Demerdash F. M., et al. 2003. | * |  | * | * | * | * | * |  | * | * | 8 |
| 12 | El-Demerdash F. M., et al. 2004. | * |  | * | * | * | * | * | * | * | * | 9 |
| 13 | Herman A., et al. 2024. | * |  | * | * | * | * | * |  | * | * | 8 |
| 14 | Jebur A., et al*.* 2023. | * |  | * | * | * | * | * | * | * | * | 9 |
| 15 | Jebur A. B., et al. 2023. | * |  | * | * | * | * | * | * | * | * | 9 |
| 16 | Khaki A., et al. 2017. | * |  | * | * | * |  | * |  | * | * | 7 |
| 17 | Khormi M. A., et al. 2024. | * |  | * | * | * | * | * |  | * | * | 8 |
| 18 | Mohamed AA-R., et al. 2023. | * | * |  | * | * | * | * | * | * | * | 9 |
| 19 | Nashed M. S., et al. 2024. | * |  | * | * | * | * | * | * | * | * | 9 |
| 20 | Osama E., et al. 2019. | * |  | * | * | * | * | * | * | * | * | 9 |
| 21 | Radi M. A., et al. 2021. | * |  | * | * | * | * | * | * | * | * | 9 |
| 22 | Rajabzadeh A., et al. 2018. | * |  | * | * | * | * | * | * | * | * | 9 |
| 23 | Sakr S. A., and Al-Amoudi W. M. 2012. | * |  | * | * | * | * | * |  | * | * | 8 |
| 24 | Soliman M. M., et al. 2025 | * |  | * | * | * | * | * | * | * | * | 9 |
| 25 | Sharma P., et al. 2012. | * |  | * | * | * | * | * | * | * | * | 9 |
| 26 | Sharma P., et al. 2014. | * |  | * | * | * | * | * | * | * | * | 9 |
| 27 | Sharma P., et al. 2016. | * | * | * | * | * | * | * | * | * | * | 10 |
| 28 | Sharma P., et al. 2018. | * |  | * | * | * | * | * | * | * | * | 9 |
| 29 | Tanushreea A. K., et al. 2024. | * |  | * | * | * | * | * |  | * | * | 7 |
| 30 | Ubah S. A., et al. 2021. | * | * | * | * | * | * | * | * | * | * | 10 |
| 31 | Vemo B. N., et al. 2017. | * |  | * | * | * | * | * | * | * | * | 9 |
| 32 | Wang H., et al. 2024 | * | * | * | * | * | * | * | * | * | * | 10 |
| 33 | Yousef M. I., et al. 2003. | * | * | * | * | * | * | * |  | * | * | 8 |
