# Supplementary material for: Natural products mitigate pyrethroid-induced male reproductive toxicity: a systematic review
Source: Front Pharmacol. 2026 Oct 26;17:1933092. doi: 10.3389/fphar.2026.1933092 (PMC13745655; doi:10.3389/fphar.2026.1933092)
Supplement: Supplementary file 3 [file Table5.docx]

Table S5: SYRCLE’s tool for assessing risk of bias

| Type of bias | Item | Domain | Review authors judgment |
| --- | --- | --- | --- |
| Selection bias | 1 | Sequence generation | Was the allocation sequence adequately generated and applied? |
|  | 2 | Baseline characteristics | Were the groups similar at baseline or were they adjusted for confounders in the analysis? |
|  | 3 | Allocation concealment | Was the allocation adequately concealed? |
| Performance bias | 4 | Random housing | Were the animals randomly housed during the experiment? |
| Performance bias | 5 | Blinding | Were the caregivers and/or investigators blinded from knowledge which intervention each animal received during the experiment? |
| Detection bias | 6 | Random outcome  assessment | Were animals selected at random for outcome assessment? |
|  | 7 | Blinding | Was the outcome assessor blinded? |
| Attrition bias | 8 | Incomplete outcome data | Were incomplete outcome data adequately addressed? |
| Reporting bias | 9 | Selective outcome reporting | Are reports of the study free of selective outcome reporting? |
| Other | 19 | Other sources of bias | Was the study apparently free of other problems that could result in high risk of bias? |
