# Supplementary material for: Natural products mitigate pyrethroid-induced male reproductive toxicity: a systematic review
Source: Front Pharmacol. 2026 Oct 26;17:1933092. doi: 10.3389/fphar.2026.1933092 (PMC13745655; doi:10.3389/fphar.2026.1933092)
Supplement: Supplementary file 4 [file Table1.doc]

| **Table S1.** Description of population, intervention, comparator, and outcome (PICOs). | |
| --- | --- |
| **Population** | Population: Adult male laboratory animals (including rats, mice, rabbits, and guinea pigs), of any eligible species or strain, exposed to pyrethroids; studies involving females or non-animal populations were excluded |
| **Intervention** | Natural products, including medicinal plants, plant extracts, essential/fixed oils, isolated phytochemicals, vitamins, and other naturally derived compounds administered to pyrethroid-exposed animals |
| **Comparison** | Pyrethroid-exposed animals receiving no natural-product intervention, vehicle/control animals, or appropriate untreated controls. |
| **Outcome** | Sperm count/concentration, motility, viability, morphology, reproductive hormones, oxidative stress/antioxidant markers, testicular histopathology, apoptosis/DNA damage, and molecular reproductive outcomes. |
| **Study design** | Experimental in vivo animal studies. |
